# Next-generation ABACUS biosensors reveal cellular ABA dynamics driving root growth at low aerial humidity

**DOI:** 10.1038/s41477-023-01447-4

**Published:** 2023-06-26

**Authors:** James Rowe, Mathieu Grangé-Guermente, Marino Exposito-Rodriguez, Rinukshi Wimalasekera, Martin O. Lenz, Kartika N. Shetty, Sean R. Cutler, Alexander M. Jones

**Affiliations:** 1 Sainsbury Laboratory, University of Cambridge, Cambridge, UK; 2 Cambridge Advanced Imaging Centre, University of Cambridge, Anatomy Building, Cambridge, UK; 3 Center for Plant Cell Biology and Institute for Integrative Genome Biology, and Department of Botany and Plant Sciences, University of California, Riverside, Riverside, CA USA; 4 Present Address: Department of Botany, University of Sri Jayewardenepura, Nugegoda, Sri Lanka

**Keywords:** Abiotic, Fluorescent proteins, Plant hormones, Plant development

## Abstract

The plant hormone abscisic acid (ABA) accumulates under abiotic stress to recast water relations and development. To overcome a lack of high-resolution sensitive reporters, we developed ABACUS2s—next-generation Förster resonance energy transfer (FRET) biosensors for ABA with high affinity, signal-to-noise ratio and orthogonality—that reveal endogenous ABA patterns in *Arabidopsis thaliana*. We mapped stress-induced ABA dynamics in high resolution to reveal the cellular basis for local and systemic ABA functions. At reduced foliar humidity, root cells accumulated ABA in the elongation zone, the site of phloem-transported ABA unloading. Phloem ABA and root ABA signalling were both essential to maintain root growth at low humidity. ABA coordinates a root response to foliar stresses, enabling plants to maintain foraging of deeper soil for water uptake.

## Abstract (web summary)

ABACUS2 Förster resonance energy transfer biosensors allow an unparalleled view of abscisic acid accumulations and depletions in living plants. Well-watered roots accumulate abscisic acid in growing cells upon shoot dehydration and this is essential to maintain root growth under low humidity.

## Main

Plant decision-making is distributed rather than centrally coordinated, but to survive and overcome stresses such as lack of water, responses must also be systemically coordinated. Abscisic acid (ABA) is a phytohormone that accumulates systemically under various local water stresses to coordinate responses over a complex and often-large morphology^1^. When roots experience low-water stress, for example, ABA closes the microscopic pores on leaves (stomata) to limit systemic water loss^2–4^. Interestingly, leaf water loss can cause changes in root growth responses and architecture: increasing transpiration genetically or through increased airflow produces larger root systems in *Arabidopsis*^5^ and low relative humidity (RH) can promote root growth in many species^6–8^. Although a molecular mechanism remains elusive, it has been proposed that ABA, acting as a dehydration signal, could be coordinating these root growth responses^5,9^. The sites of ABA biosynthesis, metabolism and translocation are the subject of intensive research, but progress has been hampered by limitations in tools to quantify accumulation and depletion of ABA on a tissue/cellular scale where regulatory decisions controlling ABA dynamics are made^1,10^. The availability of sensitive reporters, particularly Förster resonance energy transfer (FRET) biosensors, for hormones, second messengers and metabolism is revolutionizing plant development, signalling and photosynthesis research^11^. Such biosensors are powerful tools to quantify metabolites in vivo at high spatiotemporal resolution^11^, including phytohormones under changing environmental conditions^12–16^. Direct ABA FRET biosensors^13,14^ that do not require additional signalling components have broad application potential beyond ABA quantification in plant cells and subcellular compartments; for example, in ABA synthesizing pathogenic fungi^17^, in human granulocytes where ABA is a cytokine^18^, or in extracts from organisms where genetic modification is difficult using purified protein in vitro^19^. However, existing ABA FRET biosensors, ABAleons and Abscisic Acid Concentration and Uptake Sensors 1 (ABACUS1s)^13,14,20^ lack the full complement of strengths in terms of the signal-to-noise ratio or affinity required to easily quantify ABA. Therefore, we engineered next-generation ABA biosensors and deployed them to dissect cellular ABA dynamics and mobilization in response to foliar humidity stress, and to establish a systemic role for ABA in maintaining local root growth in response to a distant shoot stress.

## Results

In ABAleons and ABACUS1 biosensors, ABA sensory domains are connected by linkers to a pair of fluorescent proteins^13,14^ (FP) (Supplementary Fig. 1). The orientation and distance between these FPs determine the transfer of excitation energy via FRET from a donor FP to an acceptor FP^21^. Ligand-induced conformational changes in sensory domains alter the relative positions of the FPs, which can be detected by exciting the donor and measuring a change in relative acceptor and donor emissions, hereafter referred to as emission ratio change.

**Extended Data Fig. 1:**
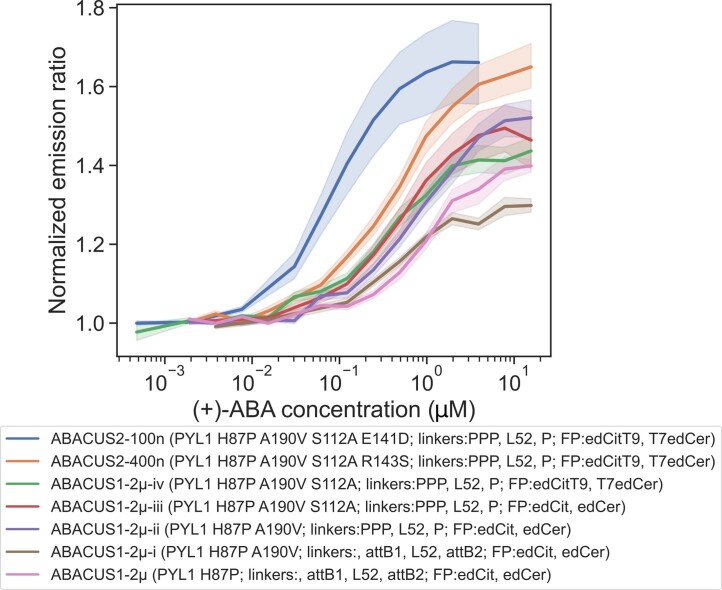
Purified ABACUS variant *in vitro* titrations. Yeast expressed ABACUS protein variants were purified with metal affinity chromatography and titrations were performed with the bioactive (+)-Abscisic acid. Emission ratio values were calculated and normalised against corresponding mock treated samples. Cit=Citrine, Cer=Cerulean. Line indicates mean of multiple biologically independent extractions and titrations, shaded region indicates the standard error of the mean. n>3 in all cases. Further details in Supplementary Table 1.

ABAleons are negative ratio change biosensors that are sensitive to endogenous ABA concentrations, but have poor signal-to-noise ratios (small emission ratio change)^13,20^. ABACUS1s have a positive ratio change with high signal-to-noise ratio but poor sensitivity for endogenous ABA^14^. Ideal biosensors are also orthogonal, with minimal interaction with endogenous signalling and vice versa. ABAleons have strong ABA hyposensitivity phenotypes, while ABACUS1s have minor ABA hypersensitivity phenotypes^13,14,20^. We used ABACUS1–2µ as the basis to engineer next-generation biosensors with high sensitivity, emission ratio change and orthogonality (Extended Data Fig. 1), screening dozens of ABACUS variants in yeast lysate or as purified proteins (Supplementary Data Tables 1 and 2).

**Fig. 1:**
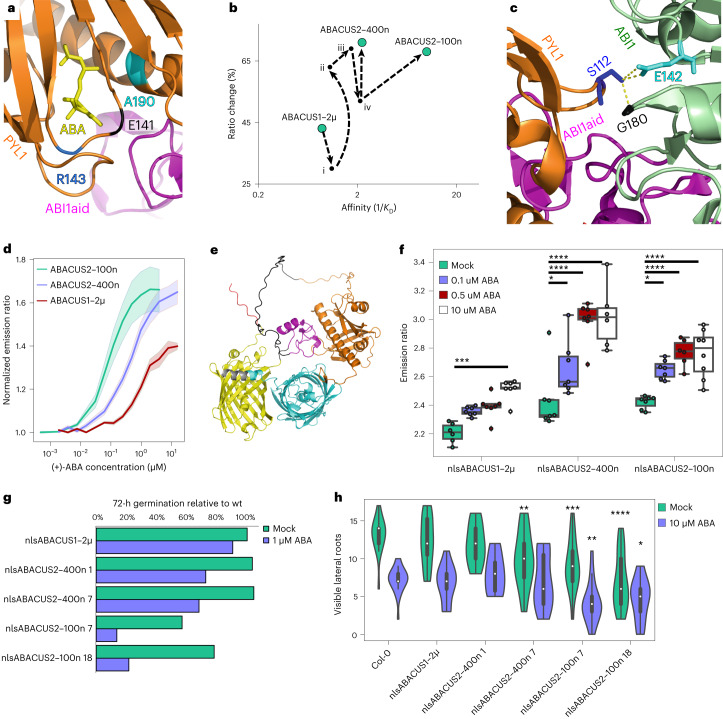
ABACUS2–100n and ABACUS2–400n offer higher ratio change and affinity than ABACUS1 but demonstrate ABA hypersensitive germination and mild to moderate root development phenotypes. **a**, Location of mutations in the PYL1–ABA and PYL-ABI1 interfaces (A190V, E141D, R143S) mapped onto crystal structure PDB: 3JRQ (ref. ^24^). **b**, Affinity vs ratio change of ABACUS variants. Intermediate sensor versions are as follows: (i) ABACUS1–2μ PYL1 H87P A190V, (ii) ABACUS1–2μ PYL1 H87P A190V, PPP-L52-P linkers, (iii) ABACUS1–2μ PYL1 H87P A190V S112A, PPP-L52-P linkers, (iv) ABACUS1–2μ PYL1 H87P A190V S112A, PPP-L52-P linkers, edCitrineT9, T7edCerulean. **c**, The ABI1-PYL1 interface. PYL1 (orange) S112 interacts with ABI1 (light green) at residues E142 and G180 but not with ABIaid (magenta). From crystal structure PDB: 3JRQ (ref. ^24^). Residue numbers correspond to their position in the wildtype proteins. **d**, Emission ratio titration of (+)-ABA for purified ABACUS variants in vitro. Line indicates mean of multiple independent extractions and titrations, and shaded regions indicate s.e.m. ABACUS1–2μ (*n* = 6), ABACUS2–400n (*n* = 16) and ABACUS2–100n (*n* = 13). **e**, Illustrative Colabfold/Alphafold MMseqs2prediction of ABACUS2–100n structure. Domains are nuclear localization signal (red), edCitrineT9 (yellow), ABI1aid(ABI1 49 aa truncation, magenta), L52 linker (black), PYL1(H87P, S112A, A190V, E141D (orange)), T7edCerulean (cyan), myc tag (grey). Structural alignment with PDB: 3JRQ of ABA-PYL1-ABI1 is available in Supplementary Fig 2. **f**, nlsABACUS emission ratio responses in *Arabidopsis* roots exposed for 30 min to various concentrations of ABA. Each point indicates median nuclear emission ratio for an individual root *z*-stack. Representative images are available in Extended Data Fig. 3. Two-way analysis of variance (ANOVA) (Sensor *F* = 64.9, *P* < 0.0001, d.f. = 2; Treatment *F* = 37.91, *P* < 0.0001, d.f. = 3; Interaction *F* = 3.349, *P* = 0.0059d.f. = 6). Asterisks indicate significance with a Dunnett post hoc test, *n* = 6, 7, 8, 7, 8, 7, 6, 6, 7, 7, 6, 8 biologically independent roots from left to right. **g**, 72 h post-stratification germination rates normalized to wildtype segregants. Full, unnormalized data are shown in Supplementary Fig. 7. **h**, Visible first-order lateral root count of ABACUS lines after treatment with 10 µM ABA. Germination was synchronized and plants grown for 6 DAG before transfer to plates with or without 10 µM ABA before quantification 6 d after transfer. Primary root growth after transfer is shown in Extended Data Fig. 4. Two-way ANOVA (Genotype *F* = 13.79, *P* < 0.0001, d.f. = 5; Treatment *F* = 94.96, *P* < 0.0001, d.f. = 1; Interaction *F* = 2.483, *P* = 0.0332d.f. = 5). Asterisks indicate significance with a Dunnett post hoc test compared to Col-0, *n* = 19, 26, 16, 14, 9, 8, 15, 18, 17, 17, 21, 18 biologically independent plants from left to right. For violin/boxplots, centre line indicates median; box limits indicate upper and lower quartiles; whiskers indicate the upper/lower adjacent values. *P < 0.05, **P < 0.01, ***P < 0.001, ****P < 0.0001.

ABACUS1–2µ has a dissociation constant for ABA (*K*_D_) of ~1.1–1.8 µM (ref. ^14^) and consists of an N-terminal FRET acceptor (edCitrine), an attB1 linker, a sensory domain consisting of a mutated pyrabactin resistant 1 like 1 (PYL1 H87P) ABA receptor, an L52 linker, a truncated protein phosphatase 2C (PP2C) co-receptor, abscisic acid insensitive 1 aba interacting domain (ABI1aid), an attB2 linker and a C-terminal FRET donor (edCerulean) (Extended Data Fig. 1)^14^. We introduced a binding site mutation into ABACUS1–2µ (PYL1 A190V (residue numbering according to position in wildtype sequence)) that is known to increase the ABA affinity of PYL1 (ref. ^22^). The resulting ABACUS1–2μ–i had increased affinity but reduced emission ratio change in vitro (Fig. 1a,b, Extended Data Fig. 1 and Supplementary Data Table 1).

Engineering increased emission ratio change is semi-empirical as mutations in any moiety may boost the transduction of ligand binding into FRET change, but a first target is often the linker between the sensory domain and the FRET pair^23^. We screened combinations of shorter, less flexible proline linkers in place of the longer, flexible attB linkers. One promising combination rescued emission ratio change of the PYL1 A190V mutant in purified protein assays (ABACUS1–2μ–ii; Fig. 1b, Extended Data Fig. 1 and Supplementary Data Tables 1 and 2).

A higher-affinity PYL1 receptor would probably exacerbate ABA hypersensitivity phenotypes of ABACUS-expressing plants through enhanced interaction with endogenous co-receptor PP2Cs (for example, ABI1, ABI2 and HAB1)^14^. Mutating PYL1 S112 disrupts PYL1 interaction with ABI1 residues E142 and G180 (ref. ^24^), residues that are absent in the ABI1aid truncation of ABACUSs (Fig. 1c). After screening for PYL1 S112 mutations that maintained emission ratio change and affinity, we selected S112A (ABACUS1–2μ–iii; Fig. 1b, Extended Data Fig. 1 and Supplementary Table 2).

We next incorrectly predicted that truncating the flexible fluorescent protein termini facing the sensory domain (edCitrine residues 1–229: edCitrineT9, edCerulean residues 8–238: T7edCerulean) would be sufficient to increase ratio change further (ABACUS1–2μ–iv; Fig. 1b, Extended Data Fig. 1 and Supplementary Data Table 1). Nonetheless, emission ratio change could be restored along with further affinity improvements by introducing either of two separate mutations (R143S, E141D) to a PYL1 region—the ‘latch’—that is important for both PYL1–ABA and PYL1–PP2C interactions^25^.

**Extended Data Fig. 2:**
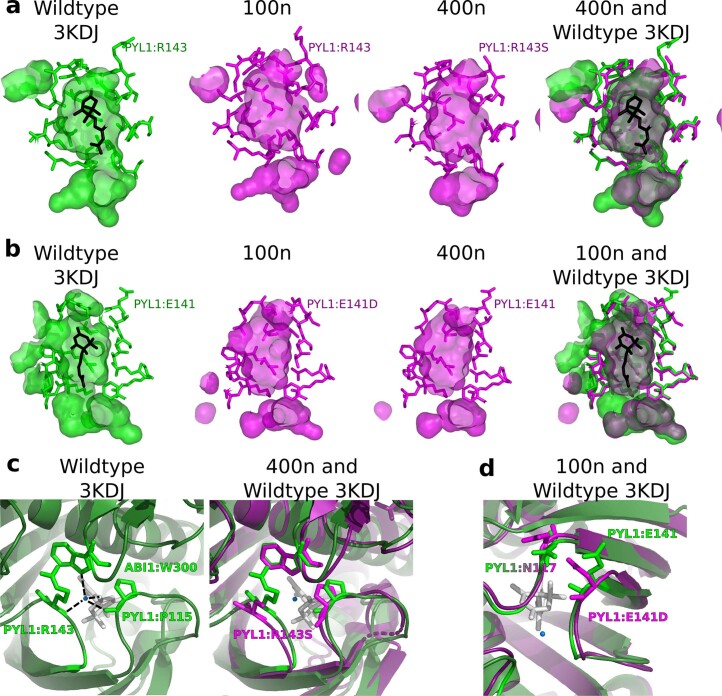
Comparisons of Colabfold predictions of the nlsABACUS2-100n and 400n binding pockets with crystal structure of PYL1-ABA-ABI1 PDB 3KDJ. **a**) Comparison of the PYL1 ABA binding pocket and latch in wildtype crystal structure 3KDJ with ABACUS2 Colabfold structural prediction. The PYL1 R143S mutation (labelled in ABACUS2-400n) prediction has altered residue positions near the top of the pocket with a relatively unchanged inner pocket. **b**) Comparison of the PYL1 ABA binding pocket and latch in wildtype crystal structure 3KDJ with ABACUS2 Colabfold structural prediction. The PYL1 E141D (labelled in ABACUS2-100n) prediction has altered residue positions near the top of the pocket with a relatively unchanged inner pocket. **c**) The water mediated interaction between PYL1 R143, PYL1 P115 and ABI W300 is not reproduced in the ABACUS2-400n Colabfold structural prediction, which includes a PYL1 R143S. Green: 3KDJ protein; White: 3KDJ ABA; Blue: 3KDJ water molecule; Magenta: nlsABACUS2-400n prediction. **d**) The PYL1 E141D mutation may alter the ‘latch’ fold. Green: 3KDJ protein; White: 3KDJ ABA; Blue: 3KDJ water molecule; Magenta: nlsABACUS2-100n prediction.

The first mutation, PYL1 E141D, inspired by sequences of the high-affinity PYL8 and PYL9 ABA receptors, produced a high ratio-change sensor with our highest affinity, which we named ABACUS2–100n (*K*_D_(ABA): 98 nM, in vitro emission ratio change: +67%; Fig. 1b,d,e and Extended Data Fig. 1). The sidechain of PYL1 E141 faces out of the ABA binding pocket (Extended Data Fig. 2 and Supplementary Fig. 2), suggesting that the high-affinity mutation could affect the accessibility of the pocket for ABA and cause faster ABA binding rather than strengthening the interaction between the pocket and ABA. Alternatively, PYL1 E141D could strengthen the PYL1 and ABI1aid interdomain interaction after ABA binding, thereby causing slower release of ABA. Introducing E141D into ABACUS1–2μ or ABACUS1–2μ–iii, which do not contain fluorescent protein truncations, did not match the affinity or ratio change of ABACUS2–100n (Supplementary Table 2). This may indicate that shortening and rigidifying the linkers between the sensory domain and the FRET pair contributed to affinity and ratio change improvements.

PYL1 R143S produced our highest ratio-change biosensor that has an ABA sensitivity suitable for in planta studies, which we named ABACUS2–400n (*K*_D_ (ABA): 445 nM, in vitro emission ratio change: +71%; Fig. 1b,d, Extended Data Fig. 1 and Supplementary Table 1). The sidechain of PYL1 R143 also faces out of the ABA binding pocket (Extended Data Fig. 2 and Supplementary Fig. 3). However, its backbone forms a water-mediated interaction with ABA, PYL1 P115 and ABI1 W300 (refs. ^24,25^). This interaction is critical as the conformational change when ABI1 W300 enters the hydrophobic pocket is known to ‘lock’ the PYL1–ABA-ABI1 intermolecular interaction during ABA signalling^24,25^. Abolishing this ‘lock’ with an ABI1 W300A mutation reduced ratio change in ABACUS1–80µ (ref. ^14^), highlighting its importance for ratio change in ABACUS biosensors. PYL1 R143S increased the ratio change in ABACUS1–2μ–iii, but as with E141D, it only increased affinity in the context of shorter and more rigid sensory domain–FRET pair linkers (Supplementary Table 2).

Alphafold2 predicts nlsABACUS2–100n and nlsABACUS2–400n structures with pockets that could still accommodate ABA (Extended Data Fig. 2). When compared with the structure of wildtype PYL1 bound to ABA^26^, the largest changes are at the pocket entrance, which is also the binding interface between PYL1 and ABI1aid moieties (Extended Data Fig. 2). Nonetheless, due to the limitations of Alphafold2 in predicting the effects of individual mutations and ligand-binding dynamics, we cannot yet discriminate which aspects of sensor behaviour are improved in these successful biosensors. For example, it remains unclear how the PYL1 E141D and R143S ‘latch’ mutations in combination with linker changes affect on- and off-rates for PYL1–ABA and PYL1–ABA–ABI1aid interactions.

Similar to ABACUS1 (ref. ^14^), in vitro assays against other phytohormones, salts and ABA-related compounds demonstrated that ABACUS2–100n is highly specific for ABA and the ABA agonist pyrabactin, although with a smaller ratio change for the latter (Supplementary Fig. 4).

Previously, severe silencing prevented us from obtaining strongly fluorescent plants expressing ABACUS1 biosensors under the control of a 641 bp *UBQ10* promoter in the *Arabidopsis thaliana* wildtype background Col-0 (ref. ^14^). Switching to the *p16* promoter, previously found to improve expression of nlsGPS1 biosensors^12^, allowed us to obtain fluorescent Col-0 plants expressing the nlsABACUS1–2μ biosensor^14^ (Supplementary Table 3). Addition of nuclear localization signals (nls) to these sensors allowed easy discrimination of the fluorescence of neighbouring cells and the exclusion of non-nuclear background and autofluorescence during image processing^11^. After screening constitutive promoters for expression of nlsABACUS2 in *Nicotiana benthamiana* transient expression assays (Supplementary Fig. 5), we selected the 1,500 bp *UBQ10* promoter^27^ and obtained strongly fluorescent *Arabidopsis* Col-0 plants expressing nlsABACUS2 biosensors (Supplementary Table 3). To accelerate ratiometric image processing for these and other nuclear targeted FRET biosensors, we developed an improved and no-cost comprehensive image analysis toolset to quickly analyse confocal stacks in three-dimensional (3D)/4D, allowing users to robustly quantify and visualize nuclear emission ratios (FRETENATOR 1.5; Supplementary Methods and ref. ^28^).

**Extended Data Fig. 3:**
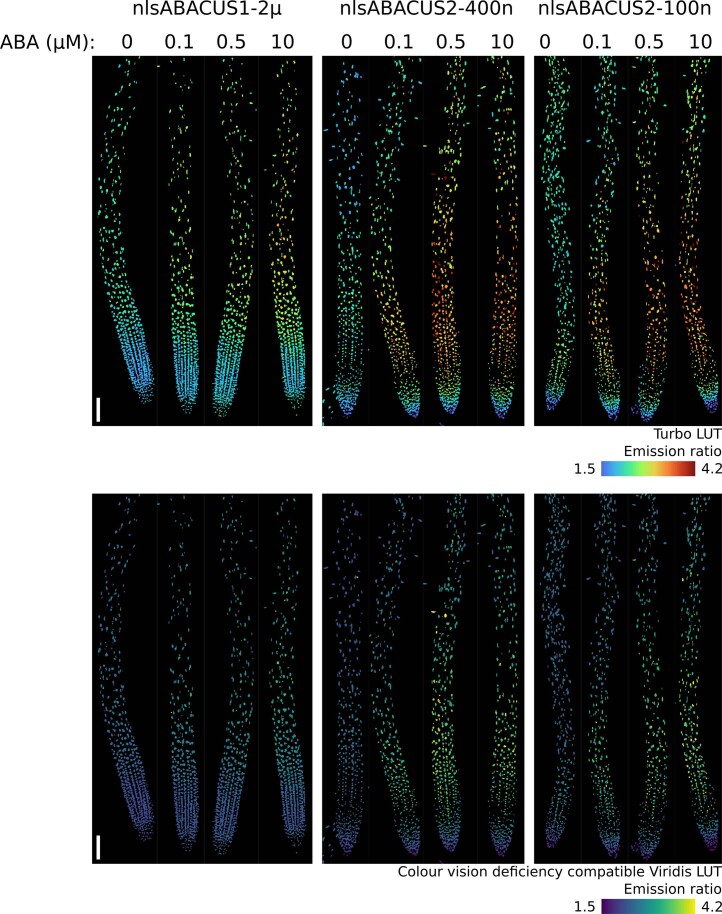
nlsABACUS2 responds to ABA *in planta*. Representative images of nlsABACUS emission ratio responses in *Arabidopsis* roots exposed for 30 minutes to various concentrations of (±)- ABA, graphed in Fig. 1f. Scale bar indicates 100 µm.

In Col-0, nlsABACUS2–400n and nlsABACUS2–100n respond strongly to exogenous ABA, saturating at lower concentrations than nlsABACUS1–2μ (Fig. 1f and Extended Data Fig. 3), consistent with their improved sensitivity. The ABACUS2 emission ratio changes are significantly larger than those of ABACUS1–2μ or other ABA sensors (ABAleonSD1-3L21) (Fig. 1f and Supplementary Data Fig. 6)^13^. Interestingly, both sensors showed a saturated response at lower concentrations of exogenous ABA than they do in vitro, perhaps indicating that the ABA import mechanisms in these cells are concentrative at these ABA treatment levels.

**Extended Data Fig. 4:**
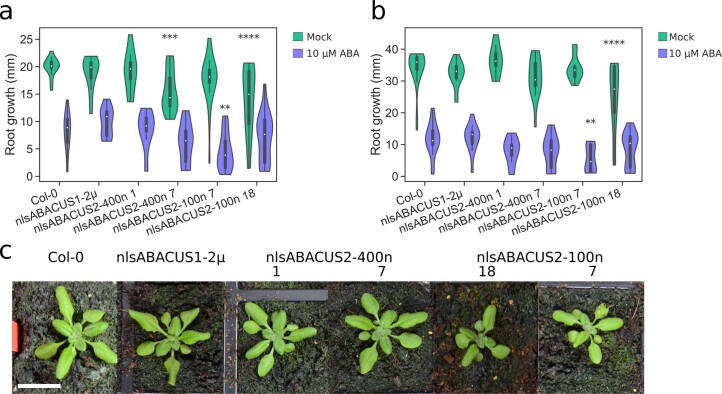
Some nlsABACUS2-400n and -100n lines display ABA hypersensitivity during growth. **a**) Primary root growth over 3 days after transfer to treatment plates at 6 DAG. Two way ANOVA (Treatment: F = 339.7 p<0.0001, DF=1; Genotype: F = 8.1, p<0.0001, DF=5; Interaction: F = 2.545, p=0.0068, DF=5). A Dunnett post hoc test was used for multiple comparisons with Col-0 within treatments. Asterisks indicate statistical significance *:p<0.05, **:p<0.01, ***:p<0.001, ****:p<0.0001, n= 22,25,17,13,9,8,24,17,23,21,20,17 biologically independent plants respectively. For violin plots, centre line indicates median; box limits indicate upper and lower quartiles; whiskers indicate the upper/lower adjacent values. **b)** Primary root growth over 6 days after transfer to treatment plates at 6 DAG. Two way ANOVA (Treatment: F = 730 p<0.0001, DF=1; Genotype: F = 5.778, p<0.0001, DF=5; Interaction: F = 4.592, p=0.0006, DF=5). A Dunnett post hoc test was used for multiple comparisons with Col-0 within treatments. Asterisks indicate statistical significance *:p<0.05, **:p<0.01, ***:p<0.001, ****:p<0.0001 n= 24,27,17,14,9,7,16,18,16,17,21,17 biologically independent plants respectively. For violin plots, centre line indicates median; box limits indicate upper and lower quartiles; whiskers indicate the upper/lower adjacent values. **c)** Representative photographs of compost grown plants at 18 days after stratification (DAS) of homozygous nlsABACUS1-µ, nlsABACUS2-400n and nlsABACUS2-100n. Numbers indicate different insertion lines. Scale bar indicated 2 cm.

To determine how the 5–25-fold higher affinity of nlsABACUS2 with orthogonalizing mutations affected ABA responses, we examined germination, lateral root development and primary root growth phenotypes known to be sensitive to ABA (Fig. 1g,h, Extended Data Fig. 4 and Supplementary Fig. 7). Without exogenous ABA, nlsABACUS2–400n lines germinate normally and nlsABACUS2–100n lines are delayed; however, all nlsABACUS2 lines display hypersensitive germination inhibition by exogenous ABA (Fig. 1g and Supplementary Fig. 7). If germination time is synchronized, primary root growth is normal in most ABACUS2 lines without exogenous ABA (Extended Data Fig. 4). However, primary root growth is hypersensitive to exogenous ABA for 3 and 6 d for nlsABACUS2–100 line 7 (Extended Data Fig. 4) and lateral root number is hypersensitive to exogenous ABA in all lines except nlsABACUS2–400n line 1 (Fig. 1h). Together, these ABA hypersensitivity phenotypes suggest that the ABACUS2 PYL1s remain somewhat active in planta despite the PYL1 S112A orthogonalizing mutation. The milder phenotypes of ABACUS2–400n expressing lines, particularly without exogenous ABA, are expected owing to their lower-affinity PYL moiety. For future investigations, using nlsABACUS2–400n (particularly line 1) may be preferable to using nlsABACUS2–100n lines if phenotypes are a concern, as long as the relevant ABA dynamics fall in the appropriate detection range.

**Fig. 2:**
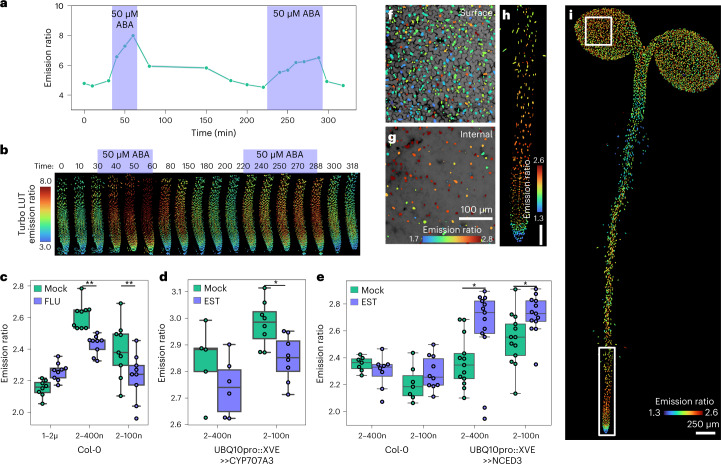
ABACUS2–100n and ABACUS2–400n reveal endogenous ABA patterns, accumulations and depletions. **a**,**b**, Graph (**a**) and maximum intensity *Z*-projection (**b**) of emission ratios of ABACUS2–400n roots responding to 50 µM exogenous ABA treatment pulses, performed with the RootChip microfluidics system. Number of nuclei in each timepoint from left to right: 996, 1,036, 996, 856, 1,020, 931, 875, 832, 935, 931, 974, 924, 931, 1,003, 932, 972, 963, 1002. **c**, 24 h fluridone treatment effect on emission ratios of nlsABACUS roots. Representative images shown in Supplementary Fig. 9. Two-way ANOVA (Treatment *F* = 7.4, *P* = 0.009, d.f. = 1; Sensor *F* = 38.0, *P* < 0.0001, d.f. = 2; Interaction *F* = 9.7, *P* = 0.0003, d.f. = 2; *n* = 8, 9, 9, 9, 9, 9 biologically independent roots from left to right). A Holm-Sidak post hoc test was used for multiple comparisons. **d**, 24 h catabolism induction (10 µM β-estradiol, UBQ10::XVE:CYP707A3) reduced nlsABACUS2–100n and nlsABACUS2–400n emission ratios in *Arabidopsis* roots. Representative images shown in Supplementary Fig 10. Two-way ANOVA (Treatment *F* = 8.1, *P* = 0.009, d.f. = 1; Sensor *F* = 9.9, *P* < 0.0046, d.f. = 1; Interaction *F* = 0.2, *P* = 0.660, d.f. = 1; *n* = 5, 6, 8, 8 biologically independent roots from left to right). A Holm-Sidak post hoc test was used for multiple comparisons. **e**, 24 h biosynthesis induction (10 µM β-estradiol, UBQ10::XVE:NCED3) increased nlsABACUS2–100n emission ratios in *Arabidopsis* roots. Representative images shown in Supplementary Fig 11. Each point indicates mean nuclear emission ratio for an individual root *z*-stack. Two-way ANOVA (Treatment *F* = 7.4, *P* = 0.008, d.f. = 1; Line *F* = 17.38, *P* < 0.0001, d.f. = 3; Interaction *F* = 2.53, *P* = 0.0637, d.f. = 3; *n* = 7, 8, 7, 10, 13, 13, 7, 10 biologically independent roots from left to right). A Holm-Sidak post hoc test was used for multiple comparisons. **f**, Surface segmentation of nlsABACUS2–400n cotyledon emission ratios. Grey to black channel represents propidium iodide counterstaining of cell walls. **g**, Internal segmentation of cotyledon emission ratios. **h**, Maximum *Z*-projection of nlsABACUS2–400n ratios in the root tip. Scale bar, 100 μm**. i**, Maximum *Z*-projection of ABACUS2–400n ratios in the cotyledons and hypocotyl. Nuclei in images have been dilated (size increased) to allow easier visual discrimination at this magnification. Boxes indicate crops of **f**, **g** and **h**. Higher resolution images with quantification and colour vision deficiency (CVD) compatible lookup tables are shown in Extended Data Fig. 6. *P < 0.05, **P < 0.01, ***P < 0.001, ****P < 0.0001. For violin/boxplots, centre line indicates median; box limits indicate upper and lower quartiles; whiskers indicate the upper/lower adjacent values.

**Extended Data Fig. 5:**
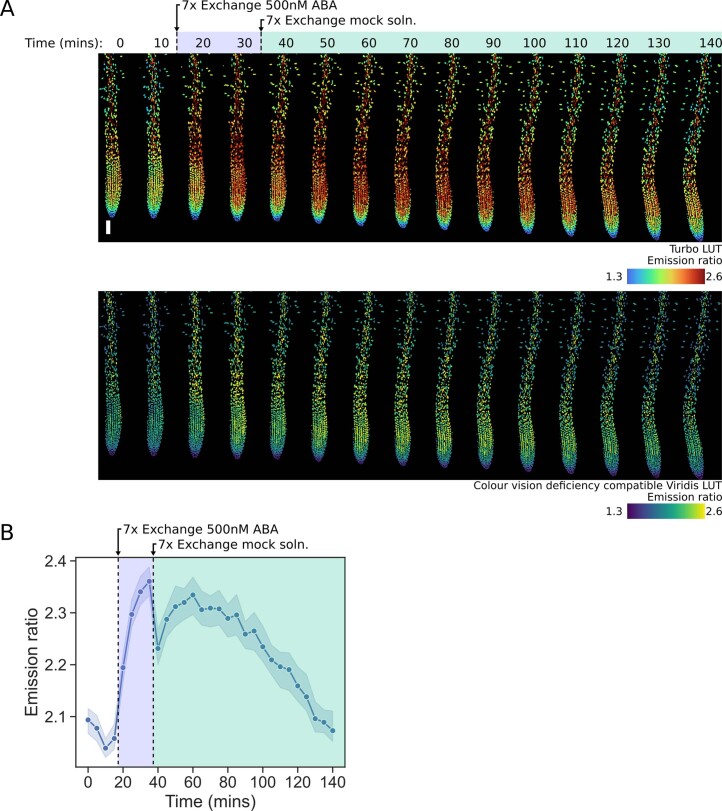
nlsABACUS2-100n is reversible *in vivo*, performed using the vacuum grease chamber buffer exchange method. **a**) Maximum intensity Z projection and graph (**b**) of emission ratios of nlsABACUS2-100n roots responding to 500 nM exogenous ABA treatment pulse. Only every second time point is shown in A to allow individual nuclei to be seen. Top: Turbo LUT, bottom: Colour vision deficiency (CVD) compatible Viridis LUT. Number of nuclei in each time point respectively: 603, 693, 677, 653, 632, 665, 658, 657, 641, 655, 611, 651, 621, 628, 648, 633, 658, 633, 620, 647, 649, 665, 665, 666, 677,657, 673, 662, 667. Scale bar indicates 100 µm. Line indicates the median. Shaded area indicates the 95% confidence interval.

As for ABACUS1–2µ, both ABACUS2 sensors were reversible in vitro and in planta, indicating that they can be used to track ABA accumulation and depletion (Fig. 2a,b, Extended Data Fig. 5 and Supplementary Fig. 8).

To determine whether the increased affinity of ABACUS2s allows them to robustly measure endogenous variations in ABA levels, unlike the lower-sensitivity ABACUS1 sensors, we undertook a pharmacological and inducible-genetics approach. The ABA biosynthesis inhibitor fluridone reduced nlsABACUS2 emission ratios, but nlsABACUS1–2μ remained level (Fig. 2c and Supplementary Fig. 9). Inducing ABA catabolism with CYTOCHROME P450, FAMILY 707, SUBFAMILY A, POLYPEPTIDE 3 (CYP707A3 (ref. ^29^)) overexpression reduced nlsABACUS2–100n emission ratios (Fig. 2d and Supplementary Fig. 10), and inducing ABA biosynthesis with 9-CIS-EPOXYCAROTENOID DIOXYGENASE 3 (NCED3) overexpression increased emission ratios (Fig. 2e and Supplementary Fig. 11). Therefore, nlsABACUS2 sensors respond to physiological levels of ABA.

**Extended Data Fig. 6:**
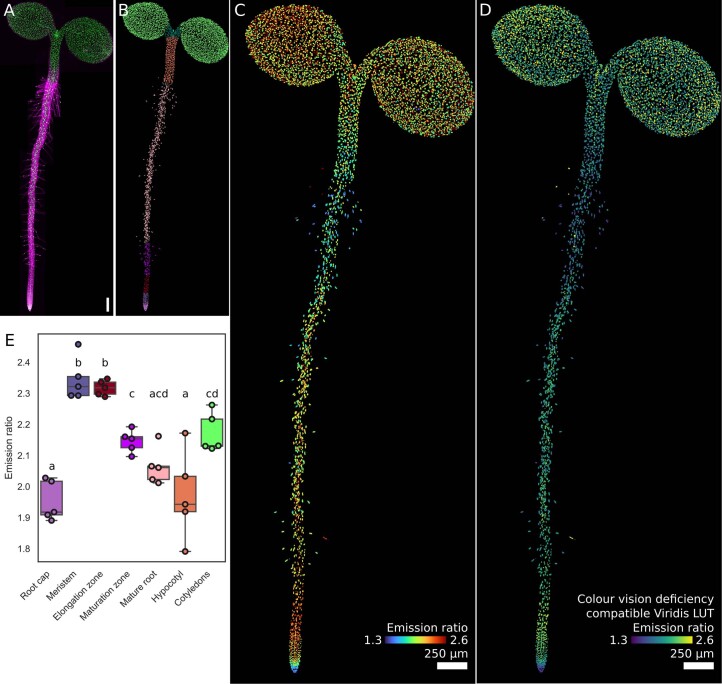
The primary root meristem and elongation zone display high ABA levels. Cotyledons and the root transition zone have intermediate ABA levels. **a**) Sum Z projection stitched micrograph of an ABACUS2-400n seedling, scale bar indicates 250 μm. **b**) Tissue labelling for quantification, corresponding to the colours in E). **c**, **d**) Max Z projection of emission ratios of seedling in (A). Note: for B), C) and D) Nuclei have been dilated (size increased) after analysis to allow easier visual discrimination at this magnification. **e**) Quantification of emission ratios in different tissues of five different plants extracted from ROI labelling in B. One way ANOVA (p<0.0001, F=21.8). Letters indicate statistical significance with a Holm-Sidak multiple comparisons test. For boxplots, centre line indicates median; box limits indicate upper and lower quartiles; whiskers indicate the upper/lower adjacent values.

The availability of sensitive reporters for other phytohormones such as auxin revolutionized plant developmental biology by revealing localized activity of a key hormone for morphogenesis and patterning^11^. Similarly, sites of ABA accumulation may give insights into developmental regulation and stress responses. Therefore, we used nlsABACUS2s to determine the distribution of ABA in whole *Arabidopsis* seedlings at the cellular scale (Fig. 2f–i, Supplementary Fig. 12 and Extended Data Fig. 6). Untreated nlsABACUS2 seedlings had high emission ratios in the root meristem and elongation zones and low emission ratios in the mature root (Extended Data Fig. 6), differences that were not apparent with the first-generation ABA sensors, possibly due to differences in affinity, localization, signal-to-noise ratio or experimental conditions^14,30^.

**Extended Data Fig. 7:**
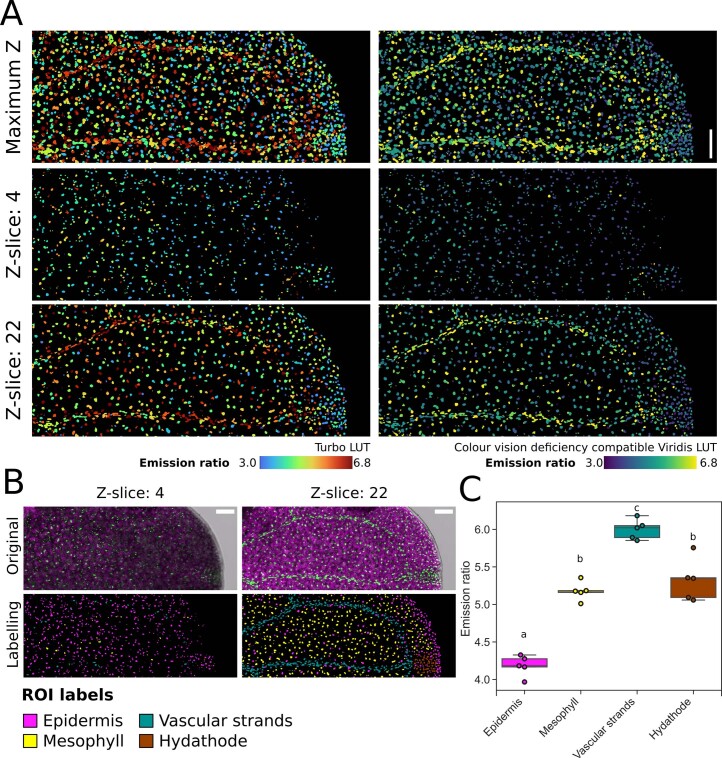
Cotyledon vascular strands display high ABA levels and the epidermis displays low ABA levels. **a**) Maximum Z projection and individual slices of nlsABACUS2-400n nuclear emission ratios from a PP11 mounted cotyledon. Z-slice 4 contains mostly epidermal cells. Z-slice 22 contains mesophyll, vascular tissues and hydathodes, as well as margin epidermis. Scale bar indicates 100μm. **b)** Original confocal Z-slices (Top) and cell type labelling (bottom) of the same cotyledon image. edCitrineT9: green; Chlorophyll: magenta; Transmitted light: grey. Scale bar indicates 100μm. **c**) Quantification of ABACUS2-400n emission ratio in epidermis, mesophyll, vascular strands and hydathode tissues. Points indicate the median value nuclear emission ratio for an individual cotyledon z-stack. One-way ANOVA (F=86.73 p<0.0001). n=5 biologically independent coltyledons. A Holm-Sidak post hoc test was used for multiple comparisons. Letters indicate statistically different groups. For boxplots, centre line indicates median; box limits indicate upper and lower quartiles; whiskers indicate the upper/lower adjacent values.

We were initially surprised that cotyledon emission ratios were not higher, as mass spectroscopy data indicate that leaves and aerial organs contain more ABA than roots in many species^9,31^. Due to the imaging modality, epidermal cells make up the bulk of segmented nuclei in our whole-plant images, but internal tissues have higher emission ratios, indicating higher ABA levels (Supplementary Fig. 13). Using PP11, an inert compound that reduces optical scattering by filling mesophyll air-spaces^32^, to image deep into cotyledons revealed that mesophyll cells have higher emission ratios than the epidermis, and vascular strands have very high emission ratios (Extended Data Fig. 7).

**Fig. 3:**
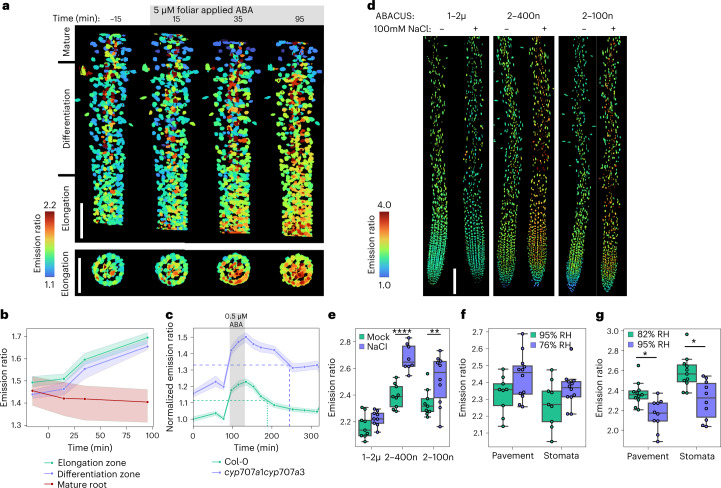
ABA accumulation in response to distal and local ABA treatments and local abiotic stresses. **a**, Max *Z*-projection and a Max *Y*-projection of emission ratios of SPIM microscopy of nlsABACUS2–400n primary root elongation and differentiation zone during a 5 µM ABA treatment to foliar tissues. Roots are isolated from the foliar tissues indicating that emission ratio increases result from ABA transport. Higher resolution images are available in Supplementary Fig 15. Scale bar, 100 µm. **b**, Quantification of different root zones shown in **a**. Points represent the median, shaded area represents the 95% confidence interval. Number of segmented nuclei at each timepoint from left to right: *n* = 570, 623, 610, 602. **c**, nlsABACUS2–400n in Col-0 and *cyp707a1cyp707a3* root tip emission ratios under ABA pulsing. Points represent the mean, shaded area represents the 95% confidence interval. Dashed lines indicate the elimination half-life (*t*_1/2_), that is, the time for emission ratio to return to half of the maximum. *n* (Col-0) = 745, 876, 736, 769, 740, 733, 737, 767, 793, 776, 841, 855; *n* (*cyp707a1cyp707a3*) = 723, 596, 596, 654, 552, 616, 557, 544, 671, 557, 583, 517 from left to right. **d**, Representative nearest-point *Z*-projection of ABACUS emission ratios in response to 6 h 100 mM NaCl treatment. Higher resolution and CVD compatible images are available in Extended Data Fig. 8. Scale bar, 100 µm. **e**, Quantification of ABACUS emission ratios in response to 6 h 100 mM NaCl treatment. Two-way ANOVA (Treatment *F* = 30.6, *P* < 0.0001 d.f. = 1; Sensor *F* = 41.02, *P* < 0.0001 d.f. = 2; Interaction *F* = 4.43, *P* = 0.017, d.f. = 2; *n* = 9, 9, 9, 8, 9, 10 biologically independent roots). A Holm-Sidak post hoc test was used for multiple comparisons. **f**, nlsABACUS2–400n emission ratios increase in response to a 6 h humidity decrease. RH indicates the measured humidity at leaf height during the treatments, which is higher than ambient chamber humidity. Representative images and peristomatal distance are available in Extended Data Fig. 9. Two-way ANOVA (Humidity *F* = 6.29, *P* = 0.0165, d.f. = 1; Cell type *F* = 2.08, *P* = 0.157, d.f. = 1; Interaction *F* = 0.0088, *P* = 0.926, d.f. = 1; *n* = 9, 12, 9, 12 biologically independent leaves from left to right). A Holm-Sidak post hoc test was used for multiple comparisons. **g**, nlsABACUS2–400n emission ratios decrease in response to a 6 h humidity increase. RH indicates the measured relative humidity at leaf height during the treatments. Representative images and peristomatal distance are available in Extended Data Fig. 10. Each point indicates median nuclear emission ratio for an individual *z*-stack. *n* = 11, 10, 11, 10 biologically independent leaves from left to right. Two-way ANOVA (Treatment *F* = 24.1, *P* < 0.0001, d.f. = 1; Cell type *F* = 13.14, *P* = 0.0008, d.f. = 1; Interaction *F* = 0.46, *P* = 0.498, d.f. = 1). A Tukey post hoc test was used for multiple comparisons. *P < 0.05, **P < 0.01, ***P < 0.001, ****P < 0.0001. For violin/boxplots, centre line indicates median; box limits indicate upper and lower quartiles; whiskers indicate the upper/lower adjacent values.

High ABA in the shoot vasculature is notable, as the phloem companion cells are a key site for ABA biosynthesis^33,34^ and ABA is thought to be transported in the phloem^9^. The phloem transports sugars, hormones and other metabolites from shoot to root, where it can be unloaded via the phloem-pole pericycle cells in the root elongation zone from two distinct vascular poles^35^. nlsABACUS2 roots show high emission ratios in the elongation zone and vascular tissues (Fig. 2h, Supplementary Fig. 14 and Extended Data Fig. 6). We used single-plane illumination microscopy (SPIM) to examine whether phloem-sourced ABA could be unloaded in the elongation zone (Fig. 3a,b and Supplementary Fig. 15). In untreated roots, nlsABACUS2–400n emission ratios were higher in two poles of the vascular tissues, as would be predicted for a phloem-transported hormone (Fig. 3b). Root emission ratios increased in the elongation zone and transition zone following shoot ABA treatment (Fig. 3a,b). The lack of mature root emission ratio increases is consistent with elongation zone ABA accumulation being sourced from the vasculature and these ABA dynamics match those of shoot-applied fluorescent dyes that are unloaded from the phloem^35^.

Exogenous ABA causes concentration-dependent promotion or inhibition of root growth^36^, so ABA from the phloem must be tightly regulated independently of local biosynthesis. The abscisic acid 8′-hydroxylases CYP707A1-4 catabolic enzymes have been implicated in eliminating ABA after stress^37,38^. *CYP707A1* and *CYP707A3* are the isoforms most expressed in the root^29^ and *cyp707a1cyp707a3* double mutants^39^ displayed a strong over-accumulation of ABA in the root tip (Supplementary Fig. 16). Exogenous ABA pulsing using the RootChip microfluidics system^12,40^ revealed larger emission ratio increases in *cyp707a1cyp707a3* and a larger elimination half-life (Fig. 3c). While these enzymes are critical in preventing over-accumulation of ABA in the root tip, other ABA depletion mechanisms must also contribute to the ABA elimination as there is still a gradual reduction in *cyp707a1cyp707a3* nlsABACUS2–400n emission ratios following an ABA pulse (Fig. 3c).

**Extended Data Fig. 8:**
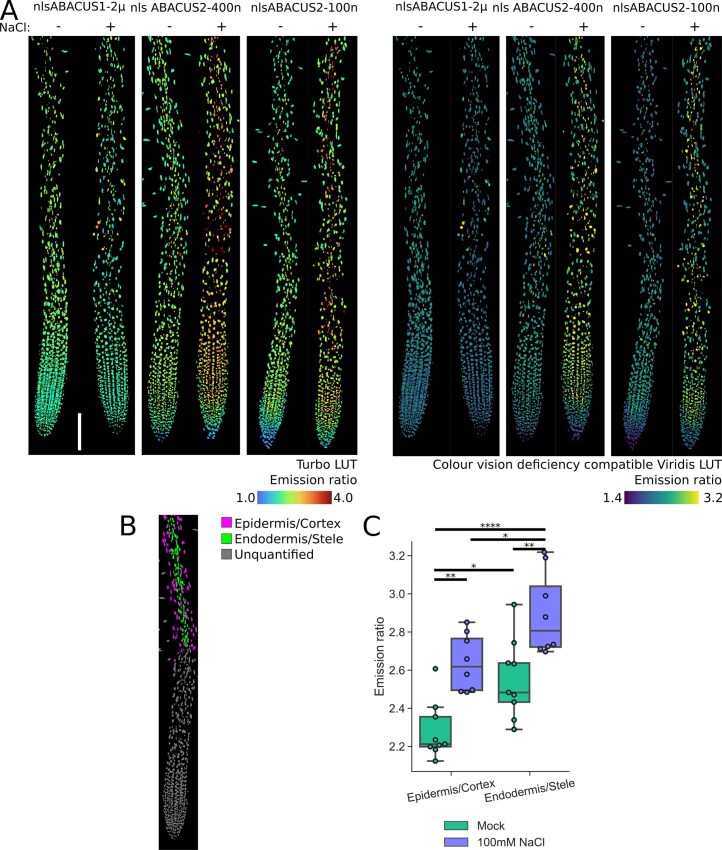
The central axis (endodermis and stele) accumulates more ABA than the epidermis and cortex under unstressed and salt conditions. **a**) ABACUS2 shows increased emission ratios following 5.5 hours of 100 mM NaCl treatment (larger reproduction of Fig. 3 f to enable tissue types to be seen). The zone used for quantification of radial ABA accumulation indicated. **b**) ROI used for quantification of nlsABACUS2-400n tissues in (C). **c**) nlsABACUS2-400n emission ratios are higher in the endodermis/stele than the epidermis/cortex in the absence and presence of 100 mM NaCl. Two way ANOVA (Tissue: F=17.38 DF=1, P=0.0002, Treatment: F=30.74 DF=1 p<0.0001, Interaction F=0.0199, DF=1, P=0.889). A Tukey post hoc test was used for multiple comparisons. Asterisks indicate statistical significance *:p<0.05, **:p<0.01, ***:p<0.001, ****:p<0.0001. n=9,8,9,8 biologically independent roots respectively. For boxplots, centre line indicates median; box limits indicate upper and lower quartiles; whiskers indicate the upper/lower adjacent values.

ABA has numerous roles in protecting plants from abiotic stress, particularly osmotic and ionic stresses. During salt stress, root ABA responses mediate endodermal cell wall suberization^41,42^, limiting ion and water flow to protect the plant; however, it is currently unclear which cells accumulate ABA. High-resolution imaging of ABACUS2s gave us an unparalleled view of the ABA accumulation after a 6 h 100 mM NaCl stress (Fig. 3d,e and Extended Data Fig. 8), allowing us to quantify which tissues accumulate ABA. Under salt stress, the stele (a site of ABA biosynthesis) and endodermis (a site of ABA-dependent protective responses) of the differentiation/maturation zones accumulate ABA to a higher concentration than the surrounding epidermis and cortex tissues (Extended Data Fig. 8).

**Extended Data Fig. 9:**
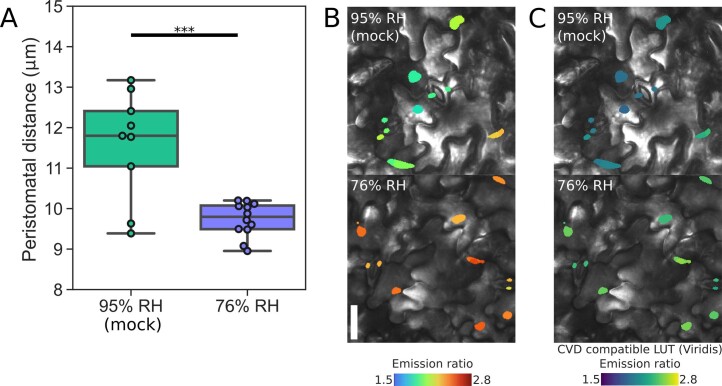
Decreasing foliar humidity increases leaf epidermal ABA levels and closes stomata. **a**) Peristomatal distance for 15 DAG nlsABACUS2-400n plants decreases following a 6-hour humidity reduction. Datapoints indicated the mean of distance of all stomata in a field of view of a confocal datastack. Each field of view contained between 8 and 16 measured stomata. Peristomatal distance taken to be a proxy for stomatal aperture^61^. Unpaired two tailed T-test p=0.0002, t=4.501, df=19. n=9,12 biologically independent leaves respectively. For boxplots, centre line indicates median; box limits indicate upper and lower quartiles; whiskers indicate the upper/lower adjacent values. **b**, **c**) Cropped representative images of nlsABACUS2-400n emission ratios in response to a 6-hour humidity decrease. Relative humidity (RH) indicates the measured humidity at leaf height during the treatments. Scale bar indicates 25 µm.

**Extended Data Fig. 10:**
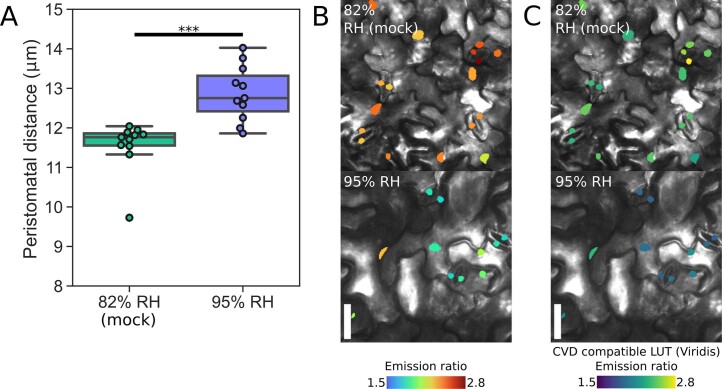
Increasing foliar humidity decreases leaf epidermal ABA levels and opens stomata. **a**) Peristomatal distance for 15 DAG nlsABACUS2-400n plants decreases following a 6-hour humidity increase. Datapoints indicated the mean of distance of all stomata in a field of view of a confocal datastack. Each field of view contained at between 11 and 20 measured stomata. Peristomatal distance taken to be a proxy for stomatal aperture^61^. Unpaired two tailed T-test p=0.0002, t=4.584, df=20. n=10,11 biologically independent leaves respectively. For boxplots, centre line indicates median; box limits indicate upper and lower quartiles; whiskers indicate the upper/lower adjacent values. **b**, **c**) Cropped representative images of nlsABACUS2-400n emission ratios in response to a 6-hour humidity increase. Relative humidity (RH) indicates the measured humidity at leaf height during the treatments. Scale bar indicates 25 µm.

Confident that we could image and detect cell type-specific ABA accumulations, we decided to investigate the effect of humidity on plant ABA levels and responses in detail. A 6 h humidity drop increased emission ratios in stomata and pavement cells expressing nlsABACUS2–400n (two-way ANOVA: Humidity factor *P* = 0.0165; Fig. 3f and Extended Data Fig. 9), which coincided with a decreased stomatal aperture (Extended Data Fig. 9). Leaf humidity increases trigger expression of ABA catabolic genes *CYP707A1* and *CYP707A3* (ref. ^38^) and nlsABACUS2–400n emission ratios decreased following a humidity increase (two-way ANOVA: Humidity factor *P* < 0.0001; Fig. 3g) and stomata opened (Extended Data Fig. 10). Remarkably, nlsABACUS2–400n emission ratios responded to humidity changes in both pavement cells and stomatal cells (Fig. 3f,g and Extended Data Figs. 9 and 10). ABA famously closes stomata and along with the vasculature, stomata have been proposed as sites of ABA biosynthesis^33,38,43^, but little attention has been paid to whether pavement cells accumulate ABA. Such broad ABA increases may indicate a systemic response that travels beyond the tissues responsible for fast local responses.

As foliar ABA levels increase following a humidity stress and foliar ABA can be transported to the root (Fig. 3a,b)^9,31^, we predicted that a local shoot stress may cause ABA accumulation in roots, affecting root growth and development. Leaf transpiration rates can affect root growth and morphology through an uncharacterized mechanism^5^; however, root plasticity is strongly ABA regulated under salt and other local water stresses^44,45^.

**Fig. 4:**
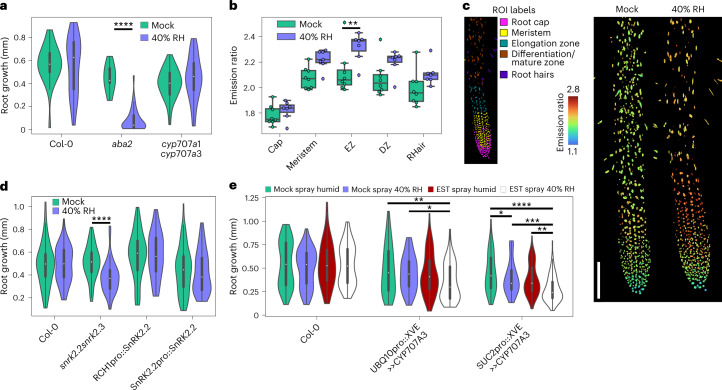
Foliar humidity decreases induce root ABA accumulation to regulate root growth. **a**, Root growth of 6 DAS Col-0, *aba2* and *cyp707a1cyp707a3* in response to 7 h foliar humidity drop, scored immediately after treatment. Two-way ANOVA (Treatment *F* = 15.0, *P* = 0.002, d.f. = 1; Genotype *F* = 31.8, *P* < 0.0001, d.f. = 2; Interaction *F* = 16.7, *P* < 0.0001, d.f. = 2; *n* = 31, 27, 36, 9, 35, 40 biologically independent roots from left to right). A Tukey post hoc test was used for multiple comparisons. **b**, Root emission ratios of nlsABACUS2–100n increase under humidity stress, with the elongation zone showing a significant ABA accumulation and the root cap showing little response. EZ, elongation zone; DZ, differentiation/maturation zone; RHair, root hair. Each point indicates the median nuclear emission ratio for an individual root *z*-stack. Two-way ANOVA (Treatment *F* = 23.64, d.f. = 1, *P* < 0.0001; Root zone *F* = 31.29, d.f. = 4, *P* < 0.0001; Interaction *F* = 0.978, d.f. = 4, *P* = 0.426; *n* = 8, 7, 8, 7, 8, 7, 8, 7, 8, 7 biologically independent roots from left to right). A Holm-Sidak post hoc test was used for multiple comparisons. **c**, Representative images of the quantification in **b**. Left: maximum *Z*-projection of ROI used to label root zones used for quantification. Right: nearest-point *Z*-projection of emission ratios at high and low humidity. Scale bar, 100 µm. **d**, Root growth of 6 DAS Col-0, *snrk2.2snrk2.3*, *snrk2.2snrk2.3* RCH1pro::SnRK2.2 and *snrk2.2snrk2.3* SnRK2.2pro::SnRK2.2 in response to a short-term foliar humidity drop, scored immediately after treatment. Two-way ANOVA (Treatment *F* = 5.158, *P* = 0.0235, d.f. = 1; Genotype *F* = 16.68, *P* < 0.0001, d.f. = 3; Interaction *F* = 4.303, *P* = 0.00052, d.f. = 3; *n* = 58, 64, 80, 60, 69, 64, 77, 52 biologically independent roots from left to right). A Holm-Sidak post hoc test was used for multiple comparisons. **e**, Root growth of 6 DAS Col-0, UBQ10pro::XVE>>CYP707A3 and SUC2pro::XVE>>CYP707A3 in response to a 7 h foliar humidity drop 24 h after a shoot spray of 50 µM β-estradiol scored immediately after humidity treatment. Two-way ANOVA (Treatment *F* = 10.91, *P* < 0.0001, d.f. = 3; Genotype *F* = 51.57, *P* < 0.0001, d.f. = 2; Interaction *F* = 3.063, *P* = 0.0057, d.f. = 6; *n* = 81, 90, 70, 70, 58, 70, 67, 68, 116, 67, 85, 111 biologically independent roots from left to right). A Holm-Sidak post hoc test was used for multiple comparisons. *P < 0.05, **P < 0.01, ***P < 0.001, ****P < 0.0001. For violin/boxplots, centre line indicates median; box limits indicate upper and lower quartiles; whiskers indicate the upper/lower adjacent values.

We developed a system where leaves could be exposed to low humidity and roots would remain hydrated (Supplementary Fig. 17) and maintain robust growth (Fig. 4a). Remarkably, the ABA biosynthesis mutant *aba2* suffered a strong root growth inhibition under low humidity (Fig. 4a), implying that ABA signalling functions to maintain root growth when foliar humidity is low, a scenario common in irrigation agriculture.

nlsABACUS2–100n roots displayed increased root emission ratios at low humidity, which were particularly prevalent in the elongation zone, the site of phloem unloading and a tissue critical for root growth (Fig. 4b,c). We took a targeted genetic approach to determine whether increases in root ABA are critical for plants to increase/maintain root growth at low humidity. ABA responses rely on the activity of the SnRK2 kinases SnRK2.2, SnRK2.3 and SnRK2.6/OPEN STOMATA 1(OST1), which phosphorylate downstream transcription factors to activate gene expression^46^. *snrk2.2snrk2.3* mutants have ABA-insensitive roots but can maintain stomatal function and closure responses under stress due to a functional SnRK2.6 protein, the principal SnRK2 responsible for phosphorylating ion channels to close stomata^47–49^.

Like *aba2*, the *snrk2.2snrk2.3* mutant demonstrated a reduced root elongation rate under humidity stress, which could be complemented with *SnRK2.2* expression under its native promoter (Fig. 4d). Complementation of the *snrk2.2snrk2.3* mutant specifically in the root meristem with RCH1pro::SnRK2.2 (ref. ^50^) allowed plants to maintain root growth under a humidity stress, indicating that local ABA signalling is required to regulate root growth as humidity varies (Fig. 4d).

ABA synthesized in the phloem companion cells^33^ is likely to be transported to phloem sinks including the root elongation zone^35^. We posited that the root induction of ABA accumulation at low foliar humidity might be phloem sourced so we performed targeted ABA depletions by controlled induction of *CYP707A3* overexpression (Fig. 4e). Regardless of whether ectopic ABA depletion was restricted to phloem-loading companion cells (SUC2pro::XVE>>CYP707A3) or was ubiquitous (UBQ10pro::XVE>>CYP707A3), root growth was inhibited at low foliar humidity (Fig. 4e). Taken together, our results indicate that phloem ABA and root tip ABA signalling regulate root growth during a distal humidity stress in leaves.

## Discussion

A series of local and systemic responses are required for plants to respond to varying water availability. Phenotypic data suggest that plant roots can respond to local osmotic differences through ABA, for example, by growing towards water (hydrotropism)^50^, but determining whether ABA levels vary across cells in a tissue has been experimentally challenging^14,20,30^. nlsABACUS2 allows ABA patterns across whole plants to be quantified at cellular resolution. nlsABACUS2 indicates that ABA is relatively low in the cotyledon epidermis, which agrees with ABAleon imaging^30^. The segmentation of nuclear localized ABACUS2 allows easier quantification of sub-epidermal tissues with reduced autofluorescence artefacts, revealing high ABA in the mesophyll and vasculature of cotyledons.

ABACUS2 sensors also reproduce the rootward transport of shoot-applied ABA, as previously demonstrated with ABAleon^13^. For basal ABA levels in roots, ABACUS1 biosensors did not indicate strong celullar patterns, presumably due to their relatively low affinity. Although ABAleons and ABACUS2 biosensors both have higher affinity, the basal ABA patterns for the two sensor families differ markedly in roots. ABAleon biosensors imply high ABA at the root–hypocotyl junction^13,30^, which decreases gradually rootward. nlsABACUS2 biosensors indicate low ABA at the root–hypocotyl junction, which increases rootward to a maximum at the meristem and elongation zone. These contrasts are potentially due to differences in biosensor properties (for example, signal-to-noise ratio and brightness), image acquisition and analysis (for example, segmentation of confocal images of nuclear targeted biosensors reduces autofluorescence artefacts), experimental conditions or developmental staging.

The high affinity, signal-to-noise ratio and nuclear localization of ABACUS2 also allowed endogenous ABA increases and decreases to be quantified at the cellular scale in shoots and roots. In a parallel study^51^, a local increase in root ABA in response to root growth through air spaces without an increase in foliar ABA levels was reported^51^. Similarly, we have shown that salt stress induces ABA accumulation in the tissues where a protective response is required—the root endodermis. However, plant roots can also induce systemic ABA accumulation. During soil drying, both sulfate and CLE25 peptides can be transported from the root to induce foliar ABA accumulation, closing stomata and limiting water loss^2–4^. During drought, some of this shoot-derived ABA is also transported down to the root to promote and maintain root growth, allowing more access to soil water^9^. That foliar tissues can sense water loss has long been known, as plants quickly regulate their stomatal aperture in response to an increased vapour pressure deficit—a process enhanced by foliar ABA accumulation^13,52^. Here we show, with cellular resolution afforded by nlsABACUS2 biosensors, that foliar drying can also regulate root ABA accumulation and that this root ABA is important to maintain root growth under stress. This demonstrates that the root and shoot can each systemically regulate each other’s responses to stresses that may only be experienced locally, providing a robust system to maintain plant water status.

## Methods

### Data visualization and statistical analysis

Unless otherwise stated, data were processed as Pandas dataframes in Python, using statsmodel/Graphpad Prism for statistics and Seaborn/matplotlib/Excel for plotting. All statistical tests are described in the figure legends, along with sample size. Statistics tables are provided in Supplementary Table 5.

### Generation of ABACUS affinity and orthogonality variants

Single amino acid mutations of the PYL1 domain of ABACUS1 in the pDRFLIP38-ABACUS1–2µ vector^14^ were performed using the QuikChange II XL (Agilent) site-directed mutagenesis kit according to manufacturer instructions. All primers used for site-directed mutagenesis are listed in Supplementary Table 4.

### Generation of ABACUS ratio-change variants

The edCitrine present in ABACUS1 variants^14^ was exchanged with a codon-diversified version for optimal expression in yeast and to allow PCR-based cloning methods. The synthetic DNA fragment containing the codon-diversified edCitrine was introduced in the ABACUS yeast expression vectors using the In-Fusion kit (Takara Bio) according to manufacturer instructions.

The poly-proline screen variants, which included substitution of the attB1 and attB2 linkers of ABACUS1 with 1–3 proline residues, and the fluorescent protein truncations were obtained using the In-Fusion kit according to manufacturer instructions. All primers used for In-Fusion cloning are listed in Supplementary Table 4.

### Fluorescence analysis and titration with (+)-ABA of protein-purified cell lysate

Yeast cell cultures (optical density (OD)_600_ ≈ 0.6) containing yeast expression vector pDRFLIP38-ABACUS1–2µ or variants were centrifuged at 4,000 *g* for 10 min, washed once in 1 ml 50 mM MOPS buffer (pH 7.4), transferred to 1.5 ml micro-centrifuge tubes and centrifuged again at 10,000 *g* for 1 min. The supernatant was discarded and 1 ml of chilled glass bead slurry (50 mM MOPS pH 7.4, 0.1% Triton X-100 and 50% v/v 0.5 mm zirconia/silica beads (Thistle Scientific)) was added to the yeast pellet inside each tube. The tubes were then vortexed at maximum power at 4 °C for 5 min. The tubes were then centrifuged at 14,000 revolutions per minute at 4 °C for 10 min. The supernatant was transferred to previously prepared HisPur cobalt spin columns (0.2 ml; Thermo Fisher). Protein purification was performed following manufacturer instructions. The subsequent first elution from the purification column was diluted in 50 mM MOPS solution. The tubes were briefly vortexed and 100 µl of diluted eluate was transferred to 96-well flat-bottom clear microplates (Greiner). A serial dilution of (+)-ABA (Cayman Chemical) was made using a 4.5 mM stock solution in ethanol and sequentially diluting it in 50 mM MOPS solution. A 50 µl volume of each (+)-ABA dilution was added to 100 µl of sensor eluate. The sample’s fluorescence emission was recorded using a SpectraMax i3x microplate reader (Molecular Devices), scanning from 470 to 550 nm after excitation at 430 nm with a bandwidth of 5 nm. Ratio was calculated by dividing emission at 525–535 nm by emission at 480–490 nm. The data produced were analysed using GraphPad Prism to determine the *K*_D_ and ratio change of each sensor, assuming the Hill function with a single binding site.

### Reversibility testing in vitro

ABACUS2 protein was purified using HisPur cobalt spin columns as before, then loaded onto Zeba spin desalting columns with mock or 100 µM ABA, according to manufacturer instructions. On the column, two washes were performed before eluting. Post elution, protein was treated with mock or 100 µM ABA and fluorescence emission was recorded on the SpectraMax i3x as above.

### Structure prediction

nlsABACUS2–100n and nlsABACUS2–400n structures were predicted (for illustrative purposes only) using the ColabFold 1.5 notebook, based on Alphafold2, using MMseqs2 for homology detection and multiple sequence alignment pairing^53^. Of the five highest-ranked predictions by pLDDT, the prediction with the best PAE scores for PYL1 and ABI1aid were used. Structural validation and confidence measures are shown in Supplementary Figs. 2 and 3.

### Cloning ABA biosynthetic and catabolic enzyme constructs for inducible expression in plants

*AtNCED3* (AT3G14440.1) was amplified with attB1/attB2 sites with q5 polymerase following manufacturer instructions and inserted into pDONR221-f1 (ref. ^54^) with a BP reaction. *AtCYP707A3* (AT5G45340.1) coding sequence with attL1/attR1 sites was synthesized in pUC19 from Genewiz. These could then be combined with *p1R4-pAtSUC2:XVE/p1R4-pUBQ10:XVE* and *p2R3a-NosT* (ref. ^55^) through a Multisite LR reaction to generate *SUC2pro::XVE>>CYP707A3, UBQ10pro::XVE>>CYP707A3* and *UBQ10pro::XVE>>NCED3* in *pHm43GW* (ref. ^56^). Gateway cloning was performed following manufacturer instructions.

### Cloning ABACUS2 constructs for expression in plants

*ABACUS2*–*100n* and *ABACUS2*–*400n* were subcloned from the yeast vectors, reverting the codon diversification of the edCitrineT9. To do this, the sensory domain to the stop codon were amplified with attB1/attB2 sites and inserted into *pDONR221-f1* with a BP reaction. *nls-edCitrineT9* was amplified from the *nlsABACUS1–2µ* plasmid^14^ and introduced into the *pENTR221-f1-ABACUS2-truncation* vectors using In-Fusion cloning (Takara) to generate *pENTR-nlsABACUS2*–*100n* and *pENTR-nlsABACUS2*–*400n*. ABACUS2 Gateway entry clones were combined with *p1R4-pUBQ10* and *p2R3a-NosT* into *pFR7m34GW* (ref. ^57^) through a multisite LR reaction. Primers are listed in Supplementary Table 4.

### Plant transformation

*Arabidopsis thaliana* plants (Columbia, Col-0 background) were transformed by the floral dip method^58^ and successful transformants were identified by FAST RED screening^59^ or hygromycin selection. Full details of the *Arabidopsis* germplasm are available in Supplementary Table 3.

### Plant growth conditions

For endpoint root imaging experiments, plants were grown under long-day conditions (110 μE, 22 °C for 18 h; 0 μE, 18 °C for 6 h).

### Salt treatment

Seeds were surface sterilized with 96% ethanol, then sown on ½ Murashige and Skoog (MS)^60^ with 0.05% MES (pH 5.7, adjusted with KOH) in 0.8% agar plates, sealed with micropore tape, then stratified for 4 d at 4 °C. Plants were grown for 5 days afer germination (DAG) before a 5.5 h treatment. Treatment consisted of transfer to ½ MS plates containing 100 mM NaCl (Merck) or fresh ½ MS with 0.05% MES (pH 5.7, adjusted with KOH) for mock.

### Fluridone treatment

Seeds were surface sterilized with 96% ethanol, then sown on ½ MS with 0.05% MES (pH 5.7, adjusted with KOH) in 0.8% agar plates, sealed with micropore tape, then stratified for 4 d at 4 °C. Plants were grown for 5 DAG before a 24 h treatment. For treatment, plants were transferred to ½ MS with 0.05% MES (pH 5.7, adjusted with KOH), containing 0.4 μM fluridone (Merck, 45511) or an ethanol mock.

### β-estradiol induction of ABA biosynthesis/catabolism

Seeds were surface sterilized with 96% ethanol, then sown on ½ MS with 0.05% MES (pH 5.7, adjusted with KOH) in 0.8% agar plates, sealed with micropore tape, then stratified for 4 d at 4 °C. Plants were grown for 5 DAG before a 24 h treatment. Treatment consisted of transfer to ½ MS with 0.05% MES (pH 5.7, adjusted with KOH), containing 10 µM β-estradiol or a dimethylsulfoxide (DMSO) mock.

### Leaf humidity treatments for leaf imaging

nlsABACUS2–400n seeds were surface sterilized with 96% ethanol, then stratified for 4 d at 4 °C in sterile deionized water before sowing on F2 Levington’s compost. Plants were grown (120 μE, 22 °C for 18 h; 0 μE, 18 °C for 6 h) for 15 DAG before humidity treatment. Plants were germinated under a clear plastic propagator lid, which was removed at 4 DAG.

To increase humidity, the chamber was set to 60% RH and humidity increased by placing a propagator lid over the plants for 6 h before imaging. Humidity and temperature were measured at leaf height above compost at ~95% RH and 22 °C for treatment, and at ~82% RH and 22 °C for mock. Humidity and temperature were measured using a BME280 sensor.

To decrease humidity, the chamber was set to 40% RH and plants were grown with a propagator lid until treatment. For treatment, compost was covered with acetate to slow evaporation and the lid was removed for 6 h before imaging. Humidity and temperature were measured at leaf height at ~76% RH and 22 °C for treatment, and at ~95% RH and 22 °C for mock. Humidity and temperature were measured using a BME280 sensor.

### Peristomatal distance measurement

Stomatal aperture is challenging to measure from confocal images, but correlates strongly with peristomatal groove distance^61^, which we measured in our nlsABACUS2–400n humidity treatment confocal stacks. The line tool in Fiji was used to measure distance using a transmitted-light channel.

### Foliar humidity treatment for root imaging

An 8 ml volume of ½ MS with 0.05% MES (pH 5.7, adjusted with KOH) in 0.8% agar was poured into a Nunc Lab-Tek II chambered coverglass (155360, Thermo Fisher) and allowed to set. Half of the agar was aseptically removed and seeds were placed on the agar next to the coverslip to allow plant roots to grow vertically between the agar and the coverslip (Supplementary Fig. 17). Chambers were sealed three times with micropore tape and stratified for 4 d and then plants were grown to 6 d post stratification in a long-day chamber. For the humidity treatment, imaging chambers were opened, a piece of folded acetate was placed over the agar to prevent direct evaporation and aerial tissues were exposed to the 40% RH, 22 °C chamber for 6 h (Supplementary Fig. 17). Mock treatment involved opening the chamber, applying a smaller piece of acetate and resealing before returning to the growth chamber. The smaller acetate application acts as a control for any mechanical perturbation, but still retains a large area for water exchange between the agar and air, so the chamber remains humid and equilibrates quickly.

### Foliar humidity treatment for root growth assays and β-estradiol pretreatment

An 80 ml volume of ½ MS with 0.05% MES (pH 5.7, adjusted with KOH) in 0.8% agar was poured into a 10 cm square plate and allowed to set. Agar (2.5 cm) was aseptically removed from one side and seeds were placed on the agar next to the back of the plate to allow plant roots to grow vertically between the agar and plate (Supplementary Fig. 17). Plates were sealed three times with micropore tape, stratified for 4 d and then plants were grown for 6 d post stratification in a long-day chamber. Immediately before treatment, the position of the primary root was marked on the plate with a razor blade and a dissecting microscope. Plants were imaged with a flatbed scanner immediately at the end of the humidity treatment, allowing growth during the treatment to be assayed.

For the humidity treatment, plates were opened, a piece of folded acetate was placed over the agar to prevent direct evaporation and plants were exposed to the 40% RH, 22 °C chamber for 7 h (Supplementary Fig. 17). Mock treatment involved opening the plates, applying a smaller piece of acetate and resealing before returning to the growth chamber. The smaller acetate application acts as a control for any mechanical perturbation, but still retains a large area for water exchange between the agar and air, so the plate remains humid and equilibrates quickly. Immediately following humidity treatment, plates were scanned with an EPSON flatbed scanner at 1,200 dpi and saved as .tif files. Root growth was then assayed with ImageJ.

For UBQ10pro/SUC2pro:XVE>>CYP707A3 induction pretreatment experiments, 24 h before humidity treatment, plates were opened, sprayed with 50 µM β-estradiol, 0.25% DMSO, 0.05% Silwett-77 or mock solution (0.25% DMSO and 0.05% Silwett-77). Excess solution was removed with a paper towel, and plates were resealed and replaced in the growth chamber.

### RootChip microfluidics treatments

The RootChip-8S device was used for ABA pulsing as described previously^12,40^. *Arabidopsis* seeds were germinated on the bottom 5 mm of 10 µl pipette tips filled with solidified growth medium (½ MS, 0.05% MES (pH 5.7, adjusted with KOH), 1% agar). After 4–7 d, pipette tip seedlings were transferred to the polydimethylsiloxane RootChip-8S device under aseptic conditions. A peristaltic pump was used (DNE; volumetric flow rate in each channel, 5 ml min^−1^) to perfuse the roots with ¼ MS (pH 5.7) liquid media. The dead volume was assessed, and it took approximately 12 min for media to pass through the tubing to reach the root, which was accounted for when plotting the ABA treatments. Imaging was performed on an inverted Leica SP8 with a ×20 dry 0.70 HC PLAN APO objective. Sequential scanning with a 448 nm laser was used to excite the edCerulean (for edCerulean and edCitrine FRET emission) and 514 nm lasers were used to excite edCitrine (for edCitrine emission, acting as an expression control). Emission settings were 460–490 nm for Cerulean and 520–550 nm for edCitrine.

### ABA hypersensitivity germination assays

Seeds were surface sterilized, placed on large agar plates with ½ MS, 0.05% MES (pH 5.7, adjusted with KOH) and 0.8% agar with or without 1 μM ABA and stratified for 4 d. After transfer to a growth chamber, a dissecting microscope was used to score germination daily. Seedling emergence from the endosperm was used to score germination.

### ABA hypersensitivity primary and lateral root growth assays

Seeds were surface sterilized, placed on large agar plates with ½ MS, 0.05% MES (pH 5.7, adjusted with KOH) and 0.8% agar vertically in a growth chamber. At 6 DAG, seedlings of approximately equal length were transferred to mock or 10 μM ABA plates. Root tip positions were marked with a pen and plates were placed vertically in the growth cabinet for 3 and 6 d before imaging on a flatbed scanner. Primary root growth was measured from the length at transfer to the root tip with the segmented line tool of Fiji. Total visible lateral root count was assayed from the scanned images of mock-treated roots at 6 d post transfer, with the multipoint tool in Fiji, including lateral roots initiating both before and after transfer.

### Confocal imaging

An upright Leica SP8-Fliman confocal microscope was used for most biosensor imaging. An inverted Leica SP8-iphox confocal microscope was used for RootChip imaging, *cyp707a1cyp707a3* imaging and PP11 cotyledon imaging. All images were acquired as *z*-stacks in 16 bit mode, with a ×10 or ×20 dry 0.70 HC PLAN APO objective. Samples were mounted in ¼ MS (pH 5.7, adjusted with KOH).

Typical settings were as follows: sequential scanning was used with the following laser/detector settings: sequence 1: 442 excitation 5–30%, HYD1: 460–500 nm, 100 gain; HYD2 525–560 nm, 100 gain. Sequence 2: 514 excitation 5–30%, HYD2 525–560 nm, 100 gain. Scan speed 400, line averaging: 2–4, bidirectional X: on, pinhole: 1 airy unit, *Z*-step size: equal to the optical section thickness, zoom: 0.75 or 1, acquisition interval: 5–10 min, pixel size: 0.49–1 µm.

### Lightsheet microscope setup

Lightsheet microscopy was performed using a custom-built laser scanning lightsheet microscope. The design is based on an openspim geometry^62^ with dual side illumination and dual side detection. Water immersion objectives were mounted horizontally (Nikon ×10, 0.3 NA for excitation, Olympus ×20 1.0 NA for detection), with the sample suspended from the top in an agarose-filled fluorinated ethylene propylene tube. For sample placement as well as for imaging, the sample can be moved between the objectives well as rotated with piezo-driven stages (Nanos LPS-30, Nanos RPS-LW20). Image stacks were acquired by moving the sample through the stationary imaging plane. Lasers (445 nm and 488 nm; Omicron LuxX 445-100, Omicron LuxX 488-200) were used for excitation and combined in an Omicron LightHub 6 with dual fibre output. The fibre output was collimated, galvo scanned (Galvo system, Thorlabs GVSM002-EC/M) and magnified, resulting in a scanned light sheet with typical full-width at half-maximum <5 μm. Two sCMOS cameras (Hamamatsu Orca Flash 4) with 6.5 × 6.5 μm^2^ pixel size were used for detection. Two motorized filter wheels (Cairn OptoSpin) with bandpass filters (Semrock FF01-480/17, Semrock FF01-532/18) allow the recording of specific fluorescence bands. The microscope was controlled by a custom software developed in LabVIEW (National Instruments). Data were streamed to disk and converted to TIFF files directly after acquisition, resulting in image voxel sizes of 1 µm^3^.

### Lightsheet imaging

The plants were grown suspended in a cut 10 μl pipette tip as in ref. ^63^ (in ½ MS, 0.05% MES (pH 5.7, adjusted with KOH) and 0.5% agarose within fluorinated ethylene propylene tubes (i.d. 0.8 mm)). They were illuminated from 2 sides while 3 fluorescent channels were recorded sequentially (Ch1: Exc 445 nm, Em 480/17; Ch2: Exc 488 nm, Em 532/18; Ch3: Exc 445 nm, Em 532/18). Typical excitation powers set in the software were 10%–50% for 445 nm excitation and 1–3% for 488 nm excitation. Camera exposure time was set to 100 ms per plane for all channels. Multiple viewpoints (60° rotation increments) were recorded for each timepoint and combined in Fiji^64^ using the Multiview reconstruction plugin^65^ before further analysis. Foliar ABA treatment was performed by pipetting 5 μM ABA onto the cotyledons, which were isolated from the roots.

### ‘FRETENATOR 1.5’ toolset development

A fast yet flexible analysis pipeline was required to analyse biosensor data. Because the biosensors used in this paper are nuclear localized, the pipeline was designed for punctate nuclear segmentation and analysis was performed on a per nucleus basis. The toolset consisted of two plugins. ‘FRETENATOR segment and ratio 1.5’ was used to segment punctate structures, perform ratio calculations and export the data as images and as a results table. ‘FRETENATOR ROI labeller’ was used to assign specific labels to the regions of interest (ROI) produced by ‘FRETENATOR Segment and ratio’ and export this information to the results table.

### Development of FRETENATOR segment and ratio v.1.5

Fiji^64^, an open-source multiplatform widely adopted ImageJ^66,67^ distribution, was chosen as platform to allow the greatest flexibility to users. All plugins were developed in Jython using CLIJ/CLIJ2 (ref. ^68^) to perform image processing directly on the graphics card. On computers with dedicated graphics cards, this allows fast analysis and modification of the segmentation settings that can be performed through a graphical user interface (Supplementary Fig. 18) with near-real-time segmentation previews. All code is freely available at https://github.com/JimageJ/ImageJ-Tools, along with installation and usage tutorial videos.

Segmentation steps are illustrated in Supplementary Fig. 19. Preprocessing consists of extracting the segmentation channel, applying a 3D difference of Gaussian filter to smooth noise and remove background. An optional tophat filter allows further background subtraction. A choice of various automatic methods or manual thresholding is then used to generate a binary map.

An optional 3D watershed is used to split objects. Because 3D watershed can cause the loss of too many nuclei ROI or shrink them below their original size, we compare the watershed to non-watershed binary maps. A map of the ‘lost nuclei’ is generated, with these lost nuclei being added back later.

A 3D connected-components analysis is used to generate a label map of the watershed nuclei. As a watershed shrinks objects, the labelled objects are dilated (on zero-value pixels only), then multiplied by the original threshold image. This provides a good segmentation with split objects without object shrinkage.

To correct account for any ‘lost nuclei’ absent from the image, a connected-components analysis is run on the ‘lost nuclei’ map to generate labels which are supplemented back onto the first label map.

Once the segmentation is complete, voxels that are saturated on either the donor excited donor emission (DxDm) or the donor excited acceptor emission (DxAm) are excluded from analysis of both channels, and the emission ratio (DxAm/DxDm) is calculated for each ROI. The segmentation is also used to quantify position, size, donor intensity, acceptor FRET intensity, acceptor intensity, pixel count and image frame for each ROI, which are exported as a results table along with file name and ROI identifiers (Supplementary Fig. 20). The following outputs are produced upon plugin completion: Threshold stack, the Label stack, Emission ratio stack, Emission ratio maximum *Z*-projection and Emission ratio nearest-point *Z*-projection. Please note, to halve the file size of exported images, emission ratio values are multiplied by 1,000 in exported image files, allowing the files to be saved as 16-bit images, instead of 32-bit images.

A log of segmentation settings is also created every time the ‘FRETENATOR segment and ratio 1.5’ plugin is run.

### Development of FRETENATOR ROI labeller

The ROI labeller is a follow-on tool for post-segmentation analysis where users can categorize the ROI in their segmented images (Supplementary Fig. 21). It currently works on single-timepoint 3D-label images, allowing users to visually assign labels to one of 10 categories. Results are either output to an existing results table or can be used to remeasure a chosen image.

### FRETENATOR software compatibility

The majority of testing was performed on a 2017 Dell desktop (Windows 10, Intel i7-6700 CPU, 3.41 GHz, 32 GB RAM, Intel HD Graphics 4000/AMD Radeon R7 450) and a 2014 Gigabyte laptop (Ubuntu, Intel i7-4710Q, 2.5 GHz Quad core, 16 GB RAM, Nvidia GTX 860M 4 gb). We also regularly use the software on Windows, Linux and Mac machines of varying ages and specifications. Considerable speed increases are present on modern hardware with fast graphics memory. Dozens of *Arabidopsis* cotyledon *z*-stacks have been tested.

### FRETENATOR 1.5 validation by comparison with Imaris 8.2

‘FRETENATOR segment and ratio 1.5’ analysis was compared to the commercial software Imaris 8.2 (https://imaris.oxinst.com/) for validation and to ensure comparable results. Buffer exchange and segmentation were performed as previously described^28,69^. Segmentation in Imaris was performed using the surfaces wizard on the AxAm channel, with background subtraction and object splitting. The XTMeanIntensityRatio Xtension was used for emission ratio calculation.

FRETENATOR 1.5 and Imaris gave extremely close results in terms of both segmentation and quantification of emission ratio (Supplementary Fig. 22). As FRETENATOR 1.5 is free, quick to use and can be installed on old, low-specification computer hardware, FRETENATOR 1.5 was used for subsequent biosensor analysis.

### Image analysis using FRETENATOR 1.5

All segmentation and labelling were performed with the ‘FRETENATOR’ plugins. Segmentation settings were optimized for each experiment but kept constant within each experiment. The AxAm channel was used for segmentation. Watershed was used for the dense nuclei of the root tip but switched off for leaf imaging. Difference of Gaussian kernel size was determined empirically due to different magnifications, resolutions and amount of noise. As a default, Otsu thresholds were used for segmentation, but in experiments where this gave poor segmentation, a manual threshold was used on the dataset (the same value for each image in the dataset).

For time courses, images were concatenated, registered using the ‘Correct 3D drift’^70^ and ‘Manual drift correction’ plugins in Fiji before analysis. To examine internal vs external tissues of cotyledons, segmentation of epidermal tissues was performed using ‘EZ-Peeler’ v.1.16 (ref. ^71^) to generate separate surface and interior image stacks. Then further analysis using a constant threshold in FRETENATOR 1.5 was performed.

For lightsheet images, viewpoints were combined in Fiji^64^ using the ‘Multiview reconstruction’ plugin^65^. Rolling ball background subtraction (Fiji: subtract background) was performed before processing with FRETENATOR 1.5.

### Reporting summary

Further information on research design is available in the Nature Portfolio Reporting Summary linked to this article.

## Supplementary information

Supplementary Information Supplementary Figs. 1–22 and Tables 1, 3 and 4.

Reporting Summary

Supplementary Tables 2 and 5 Table 2: additional ABACUS variant screening. Table 5: statistics tables.

## Data Availability

For the purpose of open access, the author has applied a Creative Commons Attribution (CC BY) licence to any author-accepted manuscript version arising from this submission. New plant lines are deposited at the Nottingham Arabidopsis Stock Centre (NASC IDs: N2111654–N2111668). Binary vectors for ABACUS2 plant transformation and ABACUS2 constructs in pENTR221*-f1* are deposited at Addgene (IDs: 203725–203728). All data are deposited at the Cambridge data repository at 10.17863/CAM.96615.

## Code availability

The FRETENATOR 1.5 image analysis toolset, as well as installation and usage instructions, are available at https://github.com/JimageJ/ImageJ-Tools.
