# Supplementary material for: Next-generation ABACUS biosensors reveal cellular ABA dynamics driving root growth at low aerial humidity
Source: Nat Plants. 2023 Jun 26;9(7):1103–15. doi: 10.1038/s41477-023-01447-4 (PMC10356609; doi:10.1038/s41477-023-01447-4)
Supplement: Supplementary file 1 — Supplementary Figs. 1–22 and Tables 1, 3 and 4. [file 41477_2023_1447_MOESM1_ESM.pdf]

nature plants

Article

https://doi.org/10.1038/s41477-023-01447-4

Next-generation ABACUS biosensors reveal cellular ABA dynamics driving root growth at low aerial humidity

In the format provided by the authors and unedited

Contents

Supplementary Fig. 1. Domain and mutation annotation of ABACUS sensors........ 2
Supplementary Fig. 2. ColabFold: AlphaFold2 using MMseqs2 predictions of nlsABACUS2-100n structure........ 3
Supplementary Fig. 3. ColabFold: AlphaFold2 using MMseqs2 predictions of nlsABACUS2-400n structure........ 4
Supplementary Fig. 4. ABACUS2-100n displays high specificity to ABA and ABA signalling agonists........ 5
Supplementary Fig. 5. UBQ10pro and p16 give good transient expression of intermediate ABACUS variant nlsABACUS1-iii........ 6
Supplementary Fig. 6. nlsABACUS2 ABA treatments........ 7
Supplementary Fig. 7 nlsABACUS2-400n and -100n display ABA hypersensitivity during germination........ 8
Supplementary Fig. 8. ABACUS2-100n and ABACUS2-400n are reversible in *in vitro* purified protein assays........ 9
Supplementary Fig. 9. Inhibiting ABA biosynthesis reduces nlsABACUS2 emission ratios........ 10
Supplementary Fig. 10. Inducing ABA catabolism reduces nlsABACUS2 emission ratios........ 11
Supplementary Fig. 11. Inducing ABA biosynthesis increases nlsABACUS2 emission ratios........ 12
Supplementary Fig. 12. nlsABACUS2s can be used to map ABA patterns *in planta.*........ 13
Supplementary Fig. 13 Internal tissues of the cotyledon have high ABA levels........ 14
Supplementary Fig. 14. The central axis (endodermis and stele) of the root differentiation zone have high ABA levels........ 15
Supplementary Fig. 15. Foliar loaded ABA accumulation in the primary root........ 16
Supplementary Fig. 16. nlsABACUS2s demonstrate that *cyp707a1cyp707a3* hyperaccumulates ABA in roots........ 17
Supplementary Fig. 17. Aerial humidity treatments with hydrated roots for root growth assays and imaging assays. 18
Supplementary Fig. 18. *FRETENATOR segment and ratio 1.5* user interface........ 19
Supplementary Fig. 19. *FRETENATOR segment and ratio 1.5* segmentation pipeline........ 20
Supplementary Fig. 20. Original image and *FRETENATOR-Segment and ratio 1.5* outputs........ 21
Supplementary Fig. 21. User interface of *FRETENATOR-ROI labeller.*........ 22
Supplementary Fig. 22. FRETENATOR 1.5 and Imaris 8.2 give similar segmentation and results........ 23
Supplemental Table 1. Emission ratios of selected purified ABACUS variants *in vitro*........ 24
Supplemental Table 2. Additional ABACUS variant screening........ 25
Supplemental Table 3. *Arabidopsis* germplasm used in this study........ 26
Supplemental Table 4. Primers used in this study........ 28

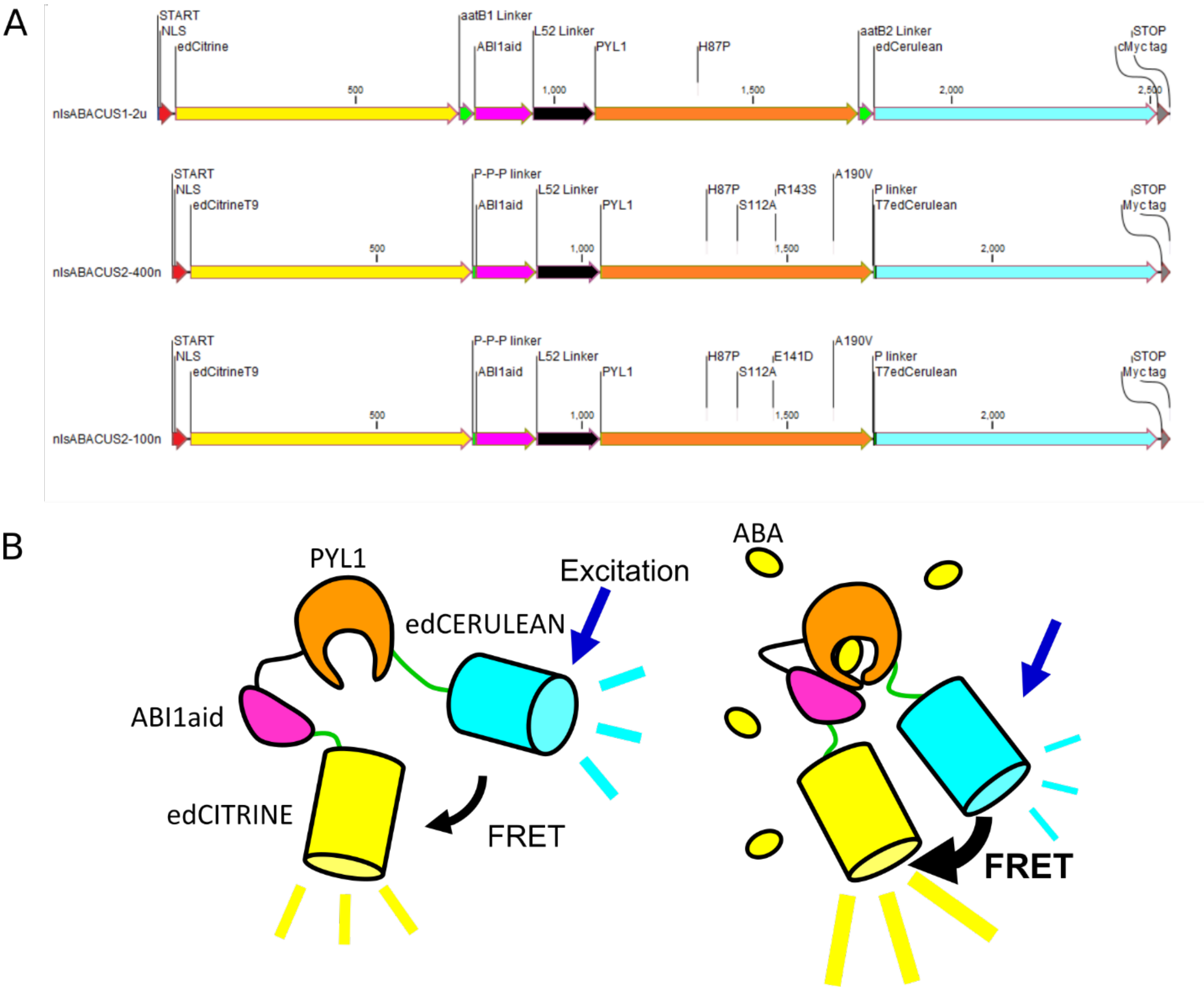

Supplementary Fig. 1. Domain and mutation annotation of ABACUS sensors.

A) Sequence annotation of nlsABACUS1-2µ, nlsABACUS2-400n (mutations: edCitrineT9, PPP-linker, PYL1: A190V, S112A, R143S, P-linker, T7edCerulean) and nlsABACUS2-100n (mutations: edCitrineT9, PPP-linker, PYL1: A190V, S112A, E141D, P-linker, T7edCerulean)

B) ABACUS cartoon representation in apo and holo forms. Upon ABA binding, there is a conformational change in the sensory domain (PYL1 and ABI1aid). The resultant change in fluorescent protein position means that more energy is transferred from the donor (edCERULEAN) to the acceptor (edCITRINE) via FRET. PPP and P linkers (green). L52 linker (black).

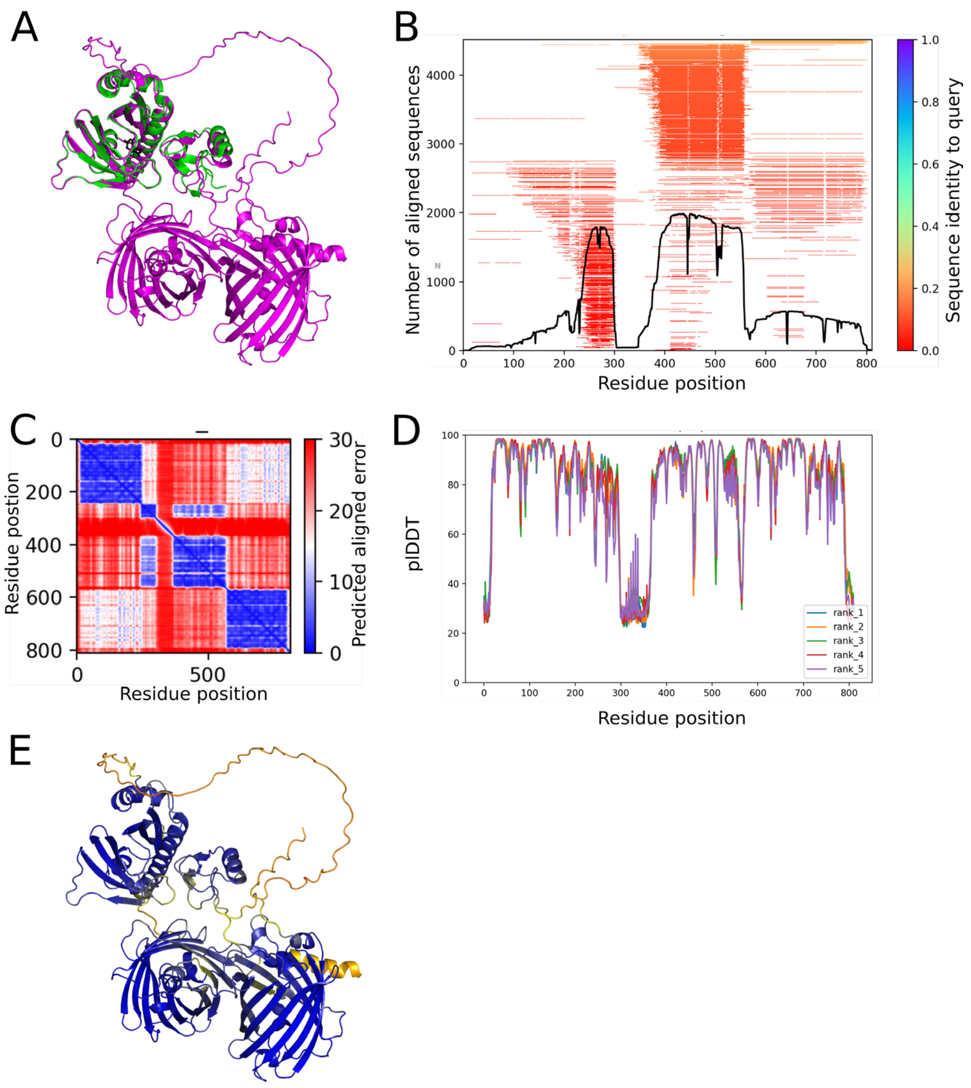

Supplementary Fig. 2. ColabFold: AlphaFold2 using MMseqs2 predictions of nlsABACUS2-100n structure

A) Highest ranked Colabfold structural prediction (magenta) of nlsABACUS2-100n aligned to PYL1-ABA-ABI1 structure 3KDJ[26] (green). Residues absent in ABACUS2 are hidden. RMSD 0.447Å (1106 to 1106 atoms).

B) Multiple sequence alignment (MSA) coverage of nlsABACUS2-100n. Black line indicates the number of aligned sequences, where very low numbers of known structures (such as in the L52 linker) give low predictive power. Coloured horizontal lines indicate the aligned sequences and their identity to the query.

C) Predicted aligned error (PAE) of the highest ranked structural prediction of nlsABACUS2-100n. Low values correspond to a high confidence in the positions of two amino acid relative to each other, making this a useful tool to determine the confidence in the relative positions between domains.

D) Predicted local distance difference test (pLDDT) plot, which uses atom positions and bond lengths to determine the confidence of local structure, shows high confidence for much of the nlsABACUS2-100n prediction.

E) pLDDT mapped onto the structural prediction, with low confidence in the nuclear localisation signal (positions 0-15), L52 spring linker (positions 296-348), the T7edCerulean C-terminus and myc-tag (791-811). Yellow: low pLDDT, Blue: high pLDDT.

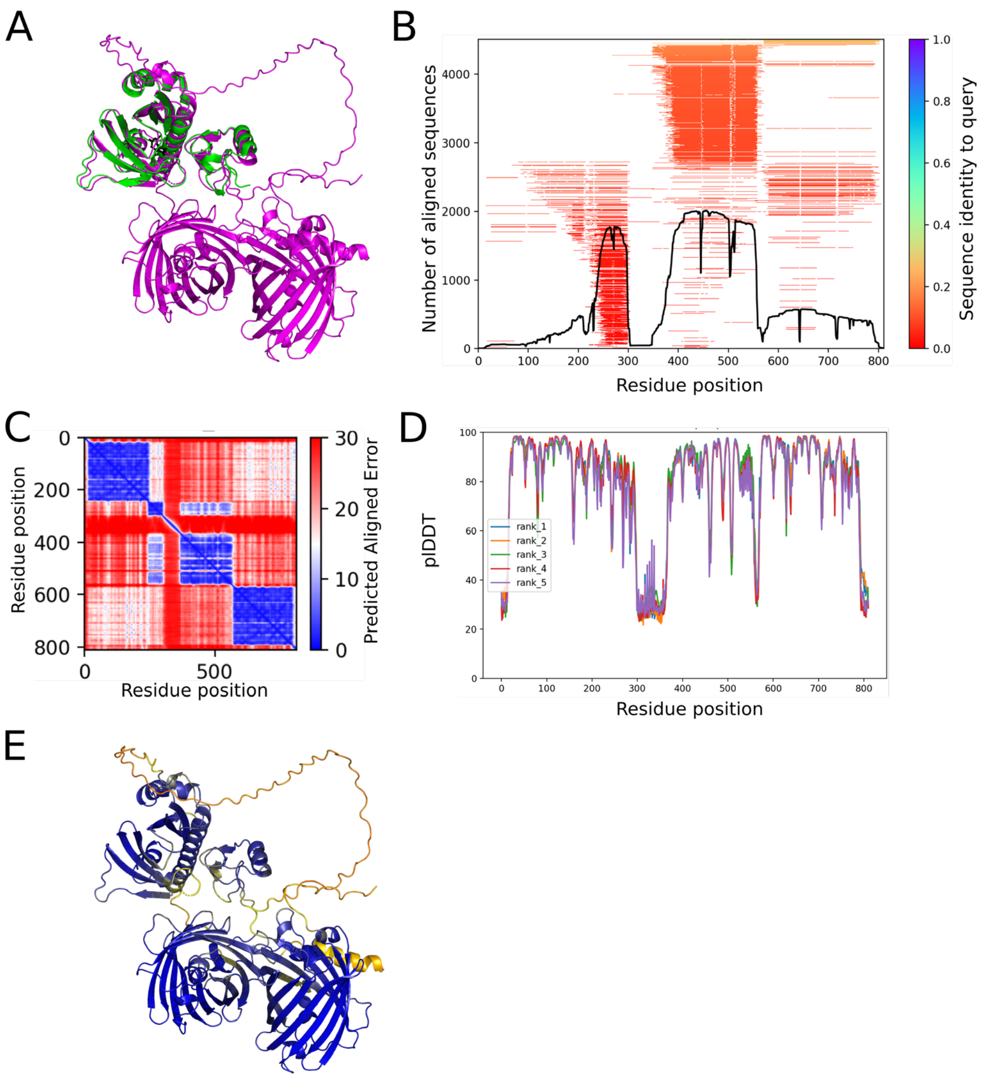

Supplementary Fig. 3. ColabFold: AlphaFold2 using MMseqs2 predictions of nlsABACUS2-400n structure

A) nlsABACUS2-400n Colabfold structural prediction (magenta) with the best Predicted aligned error (PAE) scores of the PYL1 and ABI1aid region, aligned to PYL1-ABA-ABI1 structure 3kdj (green). Residues absent in ABACUS2 are hidden. RMSD 0.464Å (1105 to 1105 atoms).

B) Multiple sequence alignment (MSA) coverage of nlsABACUS2-400n. Black line indicates the number of aligned sequences, where very low numbers of known structures (such as in the L52 linker) give low predictive power. Coloured horizontal lines indicate the aligned sequences and their identity to the query.

C) Predicted aligned error (PAE) of the same nlsABACUS2-400n prediction. Low values correspond to a high confidence in the positions of two amino acid relative to each other, making this a useful tool to determine the confidence in the relative positions between domains.

D) Predicted local distance difference test (pLDDT) plot, which uses atom positions and bond lengths to determine the confidence of local structure, shows high confidence for much of the nlsABACUS2-400n prediction.

E) pLDDT mapped onto the structural prediction, with low confidence in the nuclear localisation signal (positions 0-15), L52 spring linker (positions 296-348), the T7edCerulean C-terminus and myc-tag (791-811). Yellow: low pLDDT, Blue: high pLDDT.

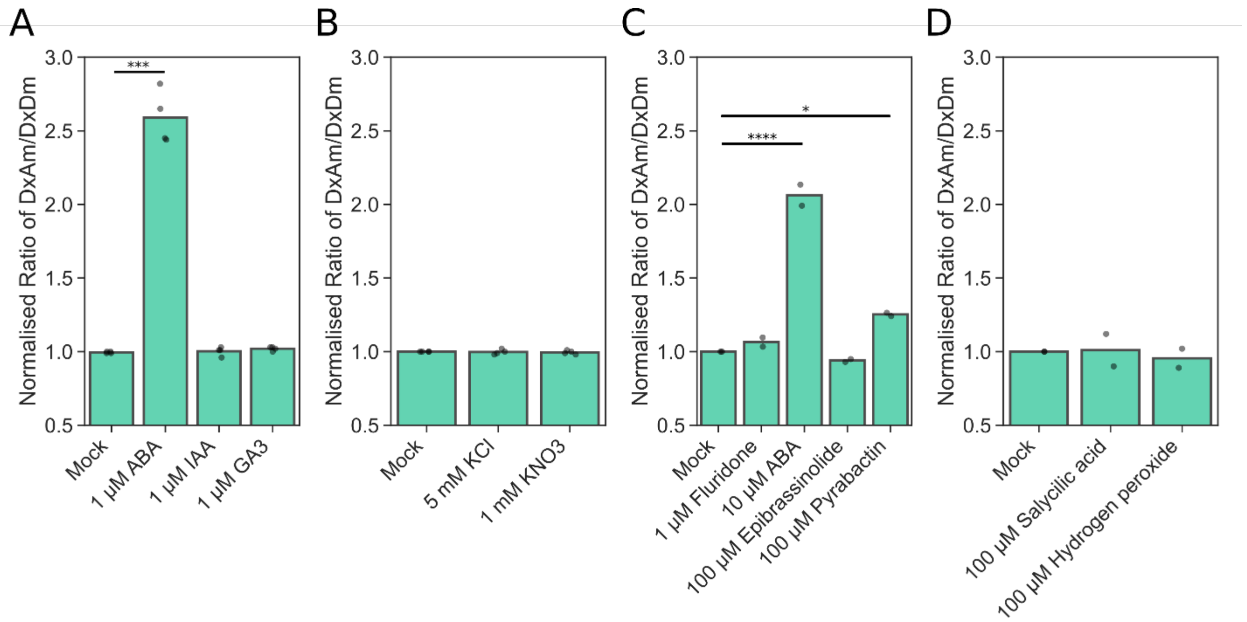

Supplementary Fig. 4. ABACUS2-100n displays high specificity to ABA and ABA signalling agonists

A) Normalised emission ratio of purified ABACUS2-100n treated with various hormones. n=4 extractions from biologically independent cultures. One-way ANOVA (F = 295.4 p<0.0001).

B) Normalised emission ratio of purified ABACUS2-100n treated with various salts. n=4 extractions from biologically independent cultures. One-way ANOVA (F = 0.1392 p=0.93).

C) Normalised emission ratio of purified ABACUS2-100n treated with ABA, the ABA biosynthesis inhibitor Fluridone, the ABA agonist Pyrabactin or the hormone Epibrassinolide. n=2 extractions from biologically independent cultures. One-way ANOVA (F = 167.6 p<0.0001).

D) Normalised emission ratio of purified ABACUS2-100n treated with Salicylic acid or hydrogen peroxide. n=2 extractions from biologically independent cultures. One-way ANOVA (F = 0.1577.6 p=0.86).

Bars indicate the mean. Asterisks indicate statistical significance with a Dunnett's post doc test. *:p<0.05, **:p<0.01, ***:p<0.001, ****:p<0.0001.

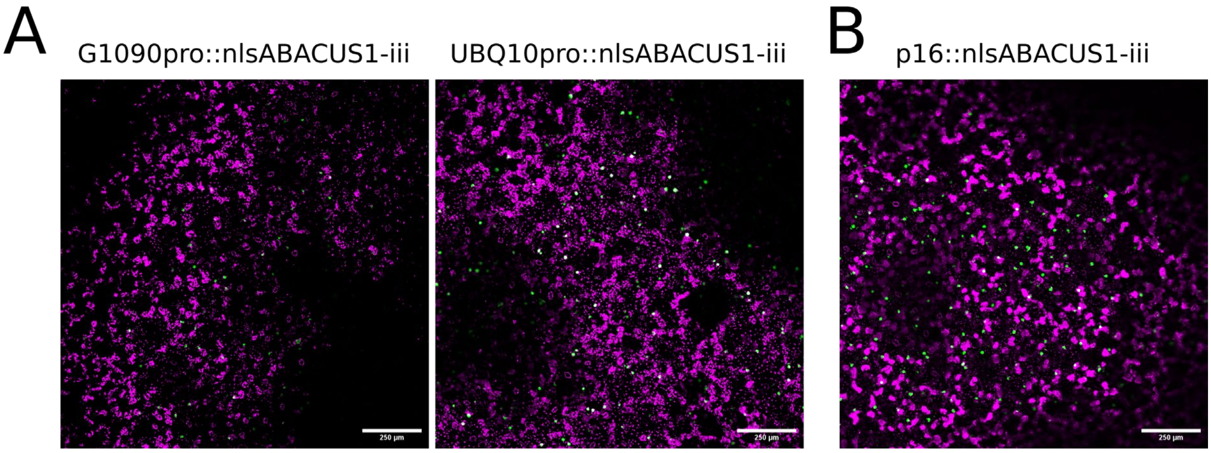

Supplementary Fig. 5. UBQ10pro and p16 give good transient expression of intermediate ABACUS variant nlsABACUS1-iii

A) *G1090pro::nlsABACUS1-iii::NosT* (n=1 infiltration) and *UBQ10pro::nlsABACUS1-iii::NosT* (n=2 independent infiltrations, repeated subsequently 3 times) both express in *Nicotiana benthamiana* transient assays, 3 days after inoculation[72].
B) *p16::nlsABACUS1-iii::NosT* gives good expression in *Nicotiana benthamiana* transient assays, 3 days after inoculation. (n=3 independent infiltrations).

Scale bars indicate 250 µm.

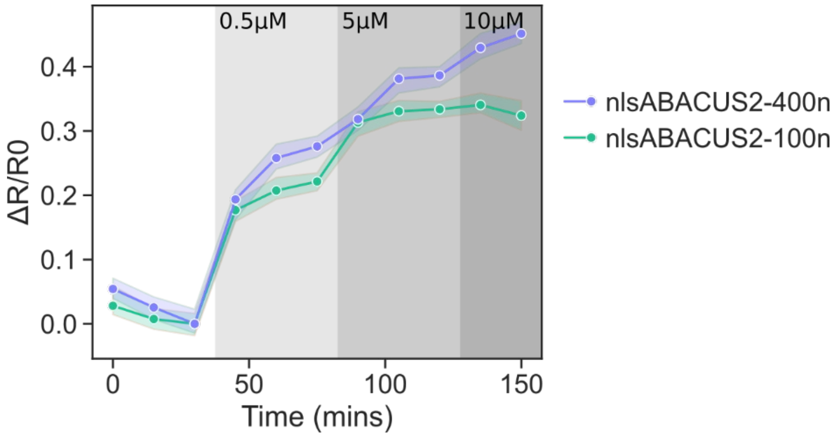

Supplementary Fig. 6. nlsABACUS2 ABA treatments.

nlsABACUS2 sensors respond to low concentrations of (±)-ABA and display a large emission ratio change in 4DAG *Arabidopsis* roots. ΔR/R0 is calculated as follows: $(Ratio_{tx}-Ratio_{t0})/Ratio_{t0}$, where $Ratio_{tx}$ is the emission ratio of a given timepoint and $Ratio_{t0}$ is the emission ratio for the timepoint immediately preceding treatment. Points indicate median, shaded area indicates 95% confidence interval.

A

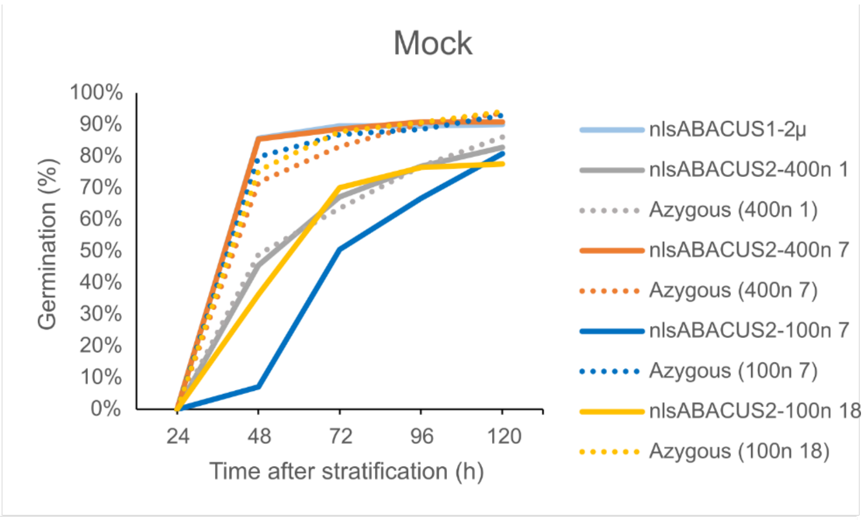

B

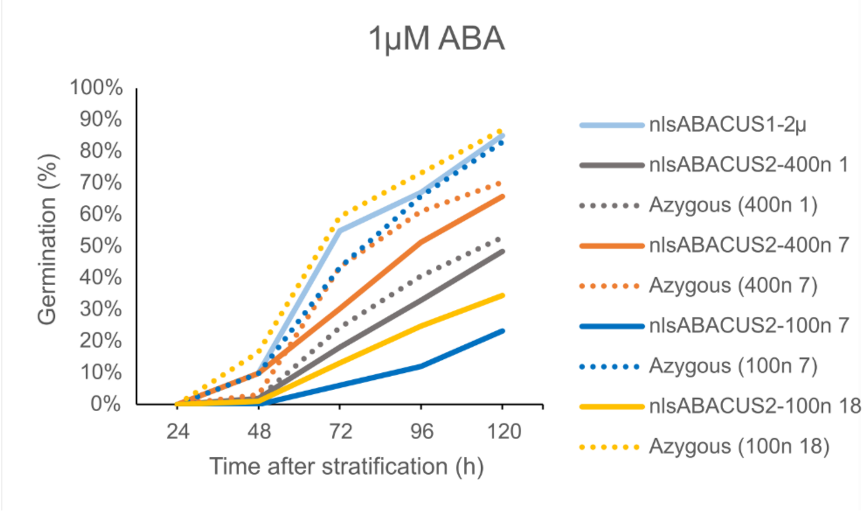

Supplementary Fig. 7 nlsABACUS2-400n and -100n display ABA hypersensitivity during germination

A) Proportion germinated (radicle/cotyledon emergence) on 1/2MS +MES plates at 24hr intervals after placing in a growth chamber. Azygous segregants from the same transformation event were grown side by side and used as wildtype controls

B) Proportion germinated (radicle/cotyledon emergence) on 1/2MS +MES 1µM ABA plates at 24hr intervals after placing in a growth chamber. Azygous segregants from the same transformation event were grown side by side and used as wildtype controls.

n= 231 (nlsABACUS1-2µ mock), 173 (nlsABACUS1-2µ ABA),151 (nlsABACUS2-400n-1 azygous mock), 197 (nlsABACUS2-400n-1 azygous ABA), 134 (nlsABACUS2-400n-1 mock),155 (nlsABACUS2-400n-1 ABA), 124 (nlsABACUS2-400n-7 azygous mock), 131 (nlsABACUS2-400n-7 azygous ABA), 219 (nlsABACUS2-400n-7 mock), 152 (nlsABACUS2-400n-7 ABA), 183 (nlsABACUS2-100n-7 azygous mock), 164 (nlsABACUS2-100n-7 azygous ABA), 99 (nlsABACUS2-100n-7 mock), 116 (nlsABACUS2-100n-7 ABA), 204 (nlsABACUS2-100n-18 azygous mock), 175 (nlsABACUS2-100n-18 azygous ABA), 187 (nlsABACUS2-100n-18 mock), 206 (nlsABACUS2-100n-18 ABA).

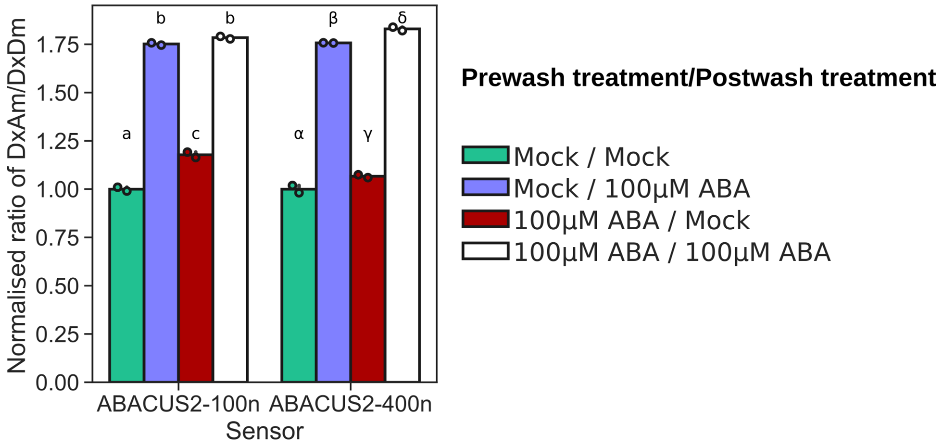

Supplementary Fig. 8. ABACUS2-100n and ABACUS2-400n are reversible in *in vitro* purified protein assays

In vitro reversibility testing of yeast expressed, purified ABACUS2-400n and ABACUS2-100n sensors, determined by fluorescent spectroscopy. Prewash treatments were performed by applying the appropriate buffer to ABACUS2 immobilised on Zeba Spin Desalting Columns. Two washes were then performed before elution. Postwash treatment was performed after elution, in plates loaded into the fluorescence plate reader. 2-way ANOVA (Sensor: $p=0.077$, $DF=1$, $F=4.115$; Treatment: $p<0.0001$, $DF=3$, $F=3242$, Interaction: $p=0.0004$, $DF=3$, $F=20.7$). Letters indicate significance groups from within sensor group Holm-Sidak multiple comparison tests.

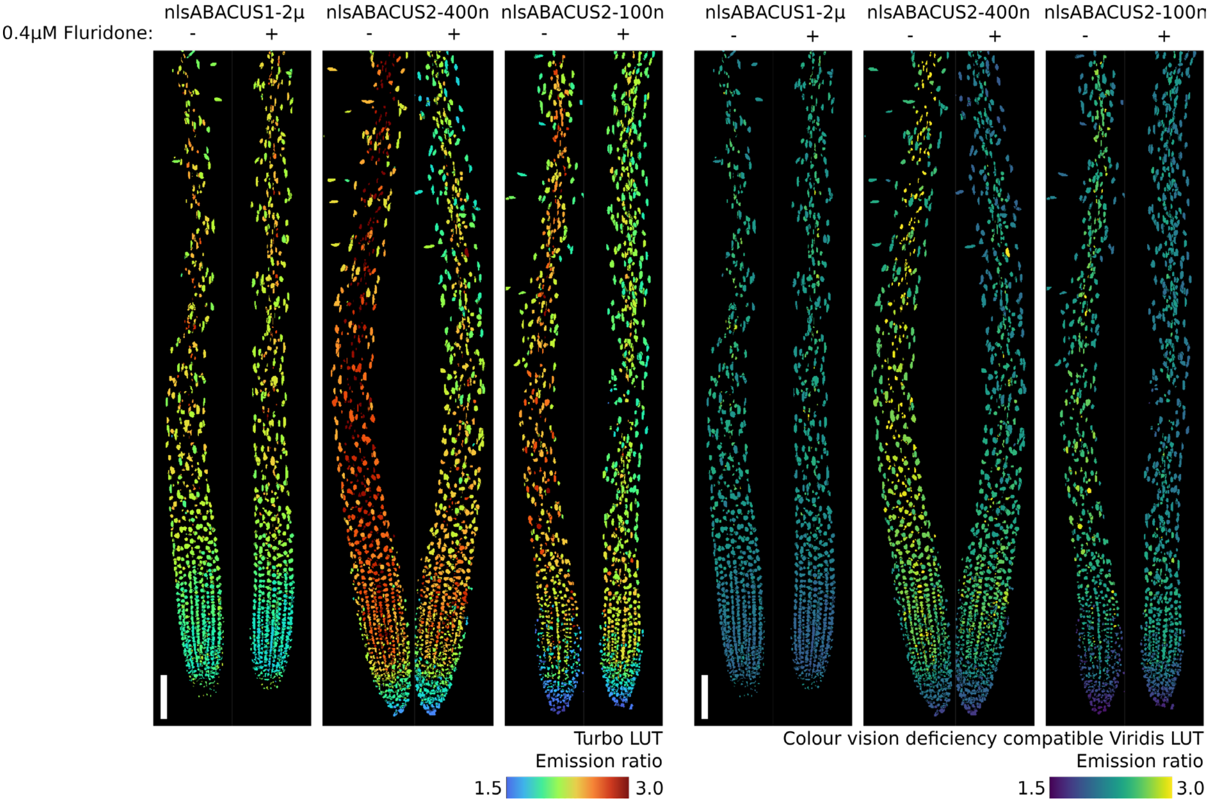

Supplementary Fig. 9. Inhibiting ABA biosynthesis reduces nlsABACUS2 emission ratios

Representative nearest point emission ratios images of nlsABACUS expressing *Arabidopsis* roots after 24hr of 0.4 µM fluridone or mock treatment. Scale bar indicates 100µm.

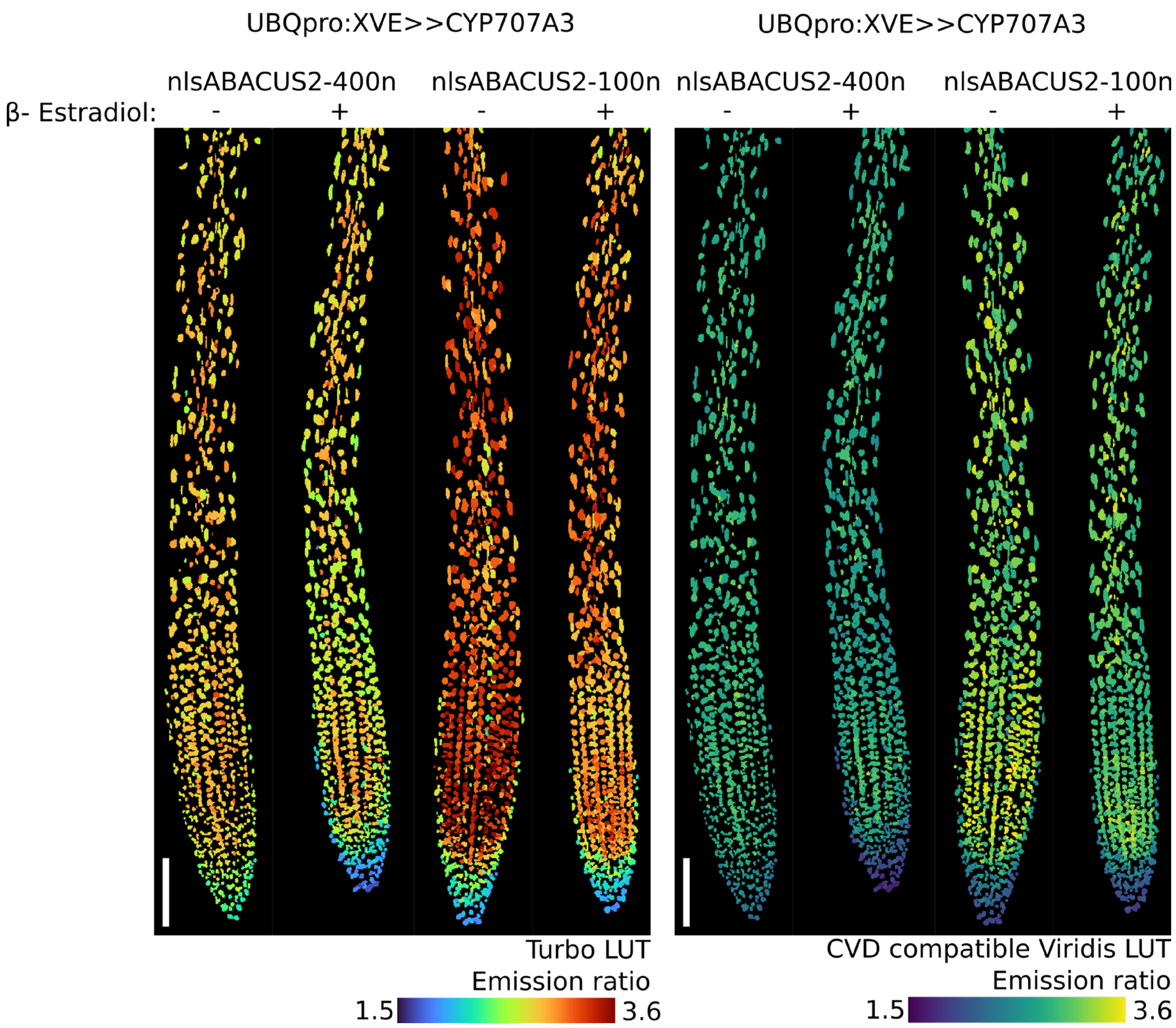

Supplementary Fig. 10. Inducing ABA catabolism reduces nlsABACUS2 emission ratios.

Representative maximum z-projection images of emission ratios in nlsABACUS2 expressing roots after 24hr mock or 10uM β-estradiol UBQ10pro:XVE>>CYP707A3 induction. Corresponds to graph of multiple roots in Fig. 2D. Scale bar indicates 100µm.

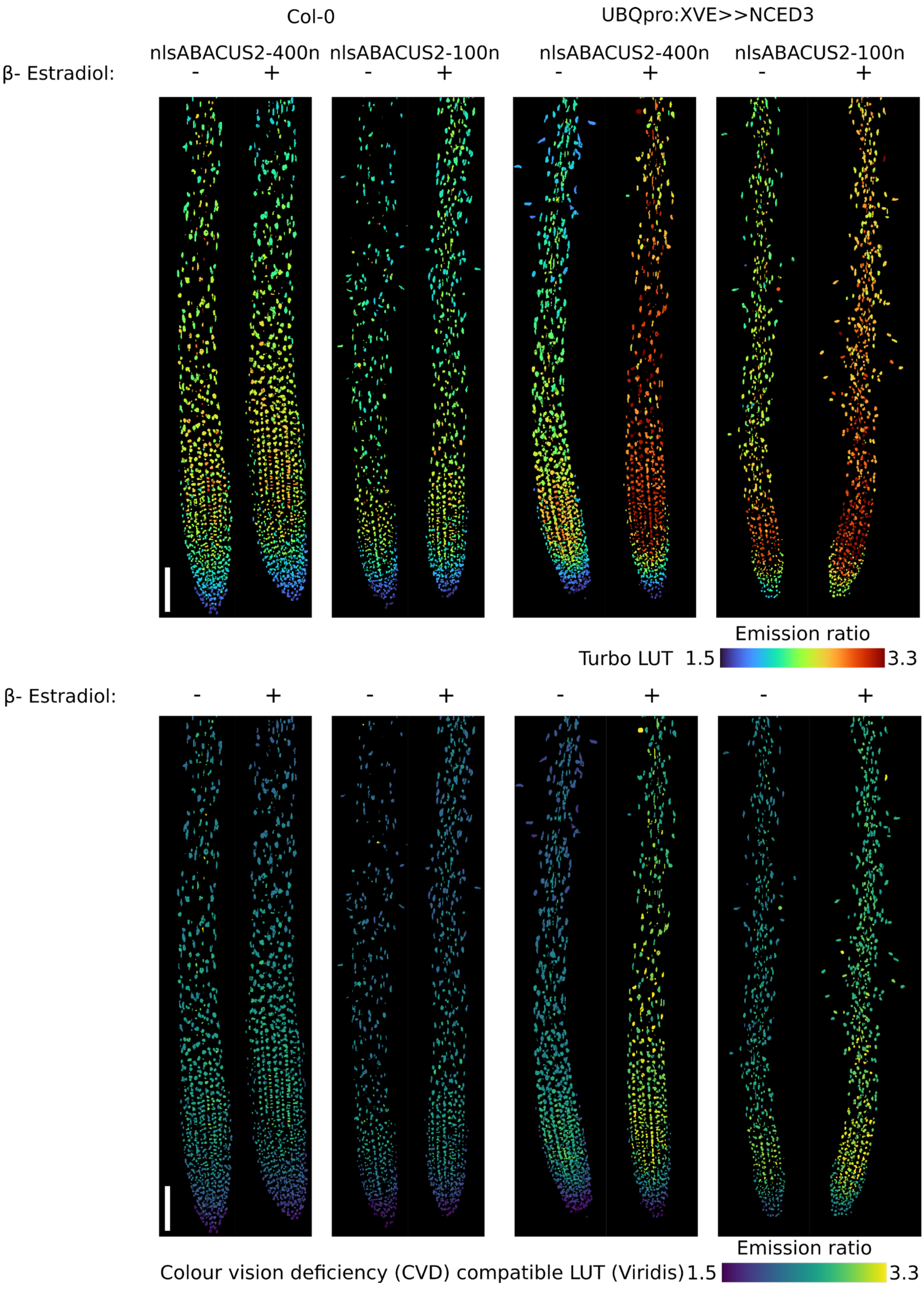

Supplementary Fig. 11. Inducing ABA biosynthesis increases nlsABACUS2 emission ratios.

Representative images of emission ratio changes in nlsABACUS2 expressing roots after 24hr mock or 10 µM β-estradiol UBQ10pro:XVE>>NCED3 induction. Corresponds to graph of multiple roots in Fig. 2E. Scale bars represent 100 µm.

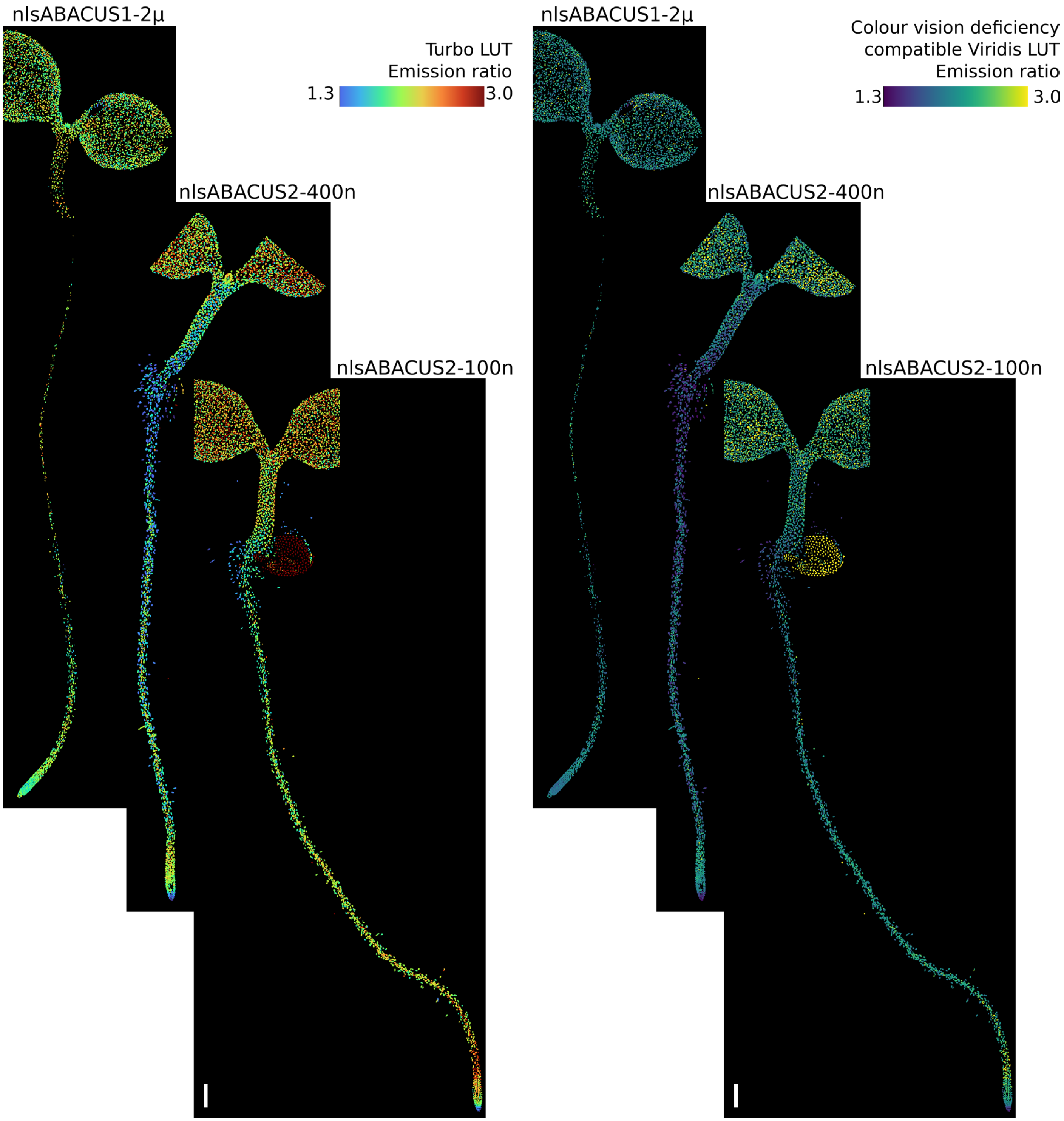

Supplementary Fig. 12. nlsABACUS2s can be used to map ABA patterns *in planta.*

nlsABACUS2 expressing *Arabidopsis* seedlings display high emission ratios in the internal tissues of the cotyledons, the vasculature and the root elongation zone/meristem/differentiation zones. Note: Nuclei have been dilated (size increased) after analysis to allow easier visual discrimination at this magnification. Scale bar indicates 250 µm.

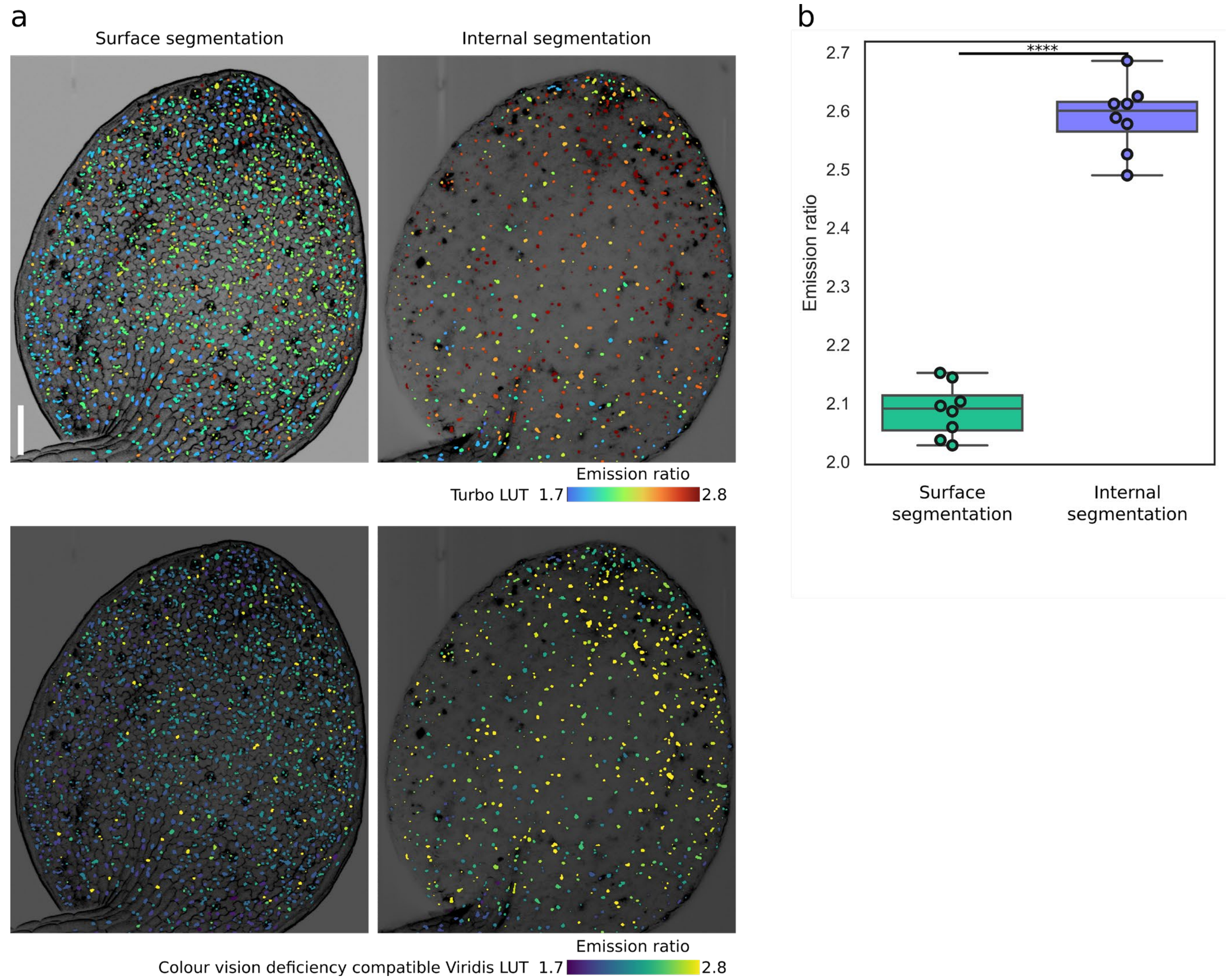

Supplementary Fig. 13 Internal tissues of the cotyledon have high ABA levels.

a) Max-Z projections of emission ratios taken from EZ-Peeler generated abaxial surface and internal segmentations of ABACUS2-400n cotyledons. Scale bar indicates 100µm. b) Quantification emission ratios for surface and internal segmentations. Note: This analysis was performed on cotyledons cropped from the same dataset of the whole plant imaging used in Extended Data Fig. 6. Two tailed T-Test (t=18.71, df=14), n=8 (2 cotyledons from 4 biologically independent plants) *:p<0.05, **:p<0.01, ***:p<0.001, ****:p<0.0001. For boxplots, centre line indicates median; box limits indicate upper and lower quartiles; whiskers indicate the upper/lower adjacent values.

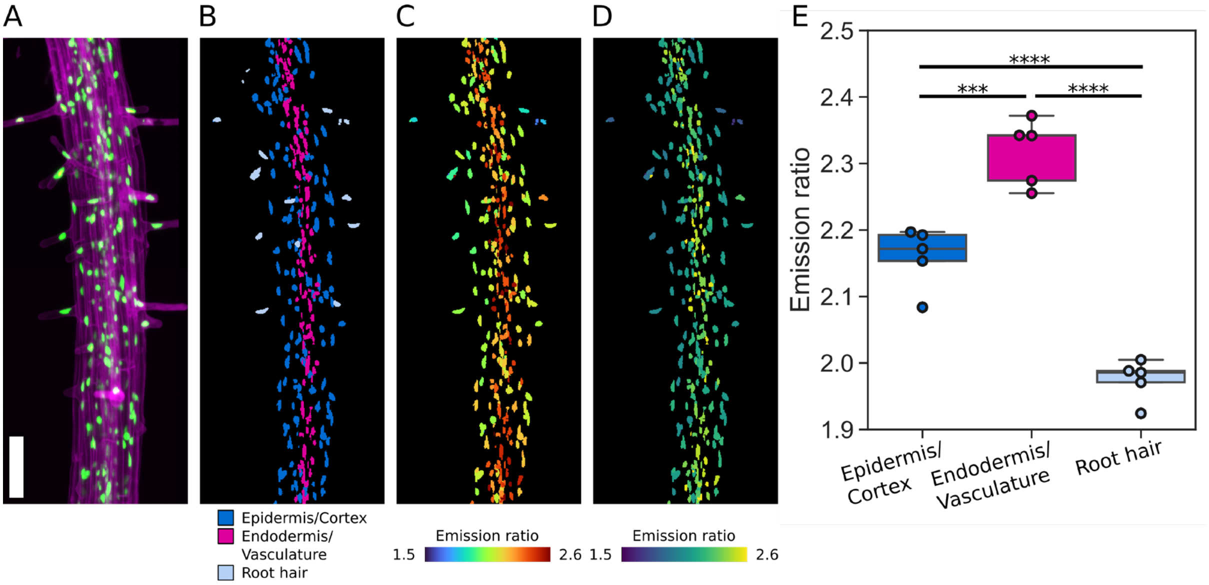

Supplementary Fig. 14. The central axis (endodermis and stele) of the root differentiation zone have high ABA levels.

A) Sum-Z projection of root nlsABACUS2-400n differentiation zone. Scale bar indicates 100µm. Green: nlsABACUS2-400n acceptor excited acceptor emission, Magenta: Propidium iodide.
B) Max-Z projection of ROI labels for different tissue types.
C) Turbo LUT and D) Colour vision deficiency compatible Viridis LUT max-Z projection of emission ratios.
E) Graph of emission ratios by tissue type. One-way ANOVA ($F = 79.86$ $p<0.0001$). $n=5$ biologically independent roots. A Tukey post hoc test was used for multiple comparisons. Asterisks indicate statistical significance *:$p<0.05$, **:$p<0.01$, ***:$p<0.001$, ****:$p<0.0001$. Note: This analysis was performed on roots cropped from the same dataset of the whole plant imaging used in Extended Data Fig. 6. For boxplots, centre line indicates median; box limits indicate upper and lower quartiles; whiskers indicate the upper/lower adjacent values

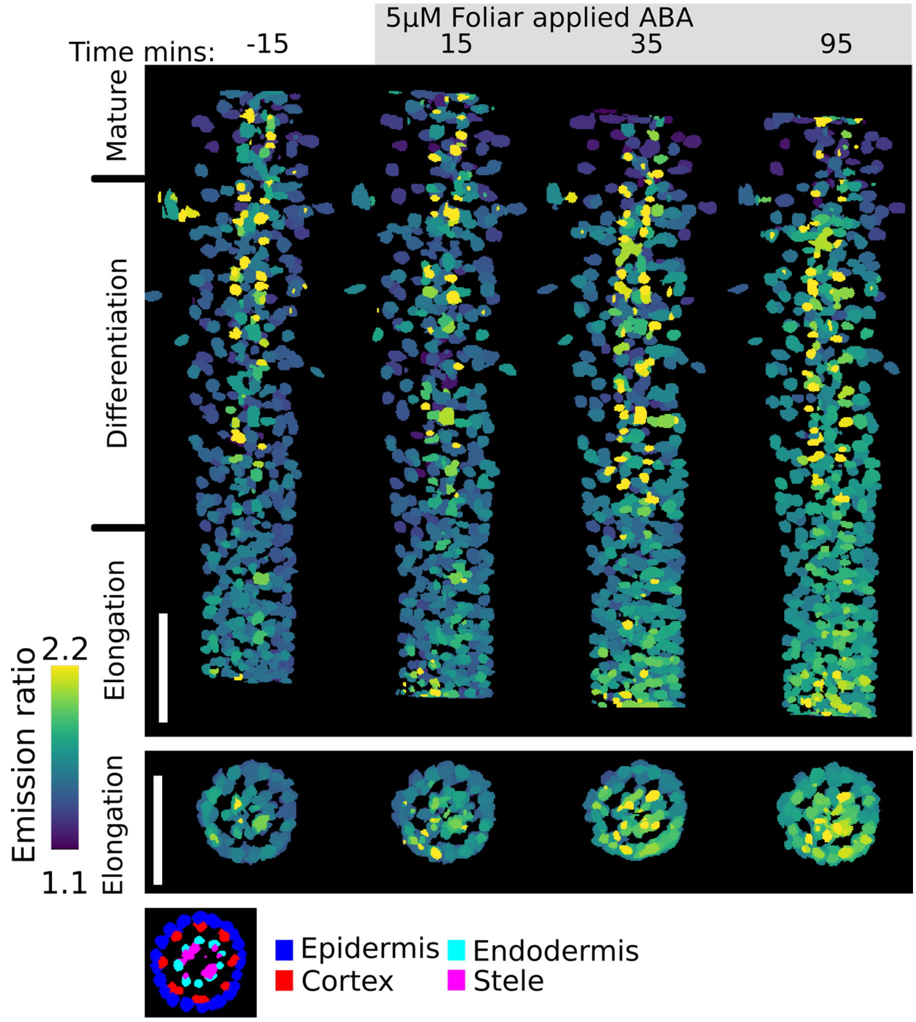

Supplementary Fig. 15. Foliar loaded ABA accumulation in the primary root.

Max Z projection of entire image and a Max Y projection of elongation zone emission ratios, captured using SPIM microscopy of nlsABACUS2-400n primary root elongation and differentiation zone during a 5µM ABA treatment to the foliar tissues. Roots are isolated from the foliar tissues indicating emission ratio increases result from ABA transport. Scale bars indicate 100µm.

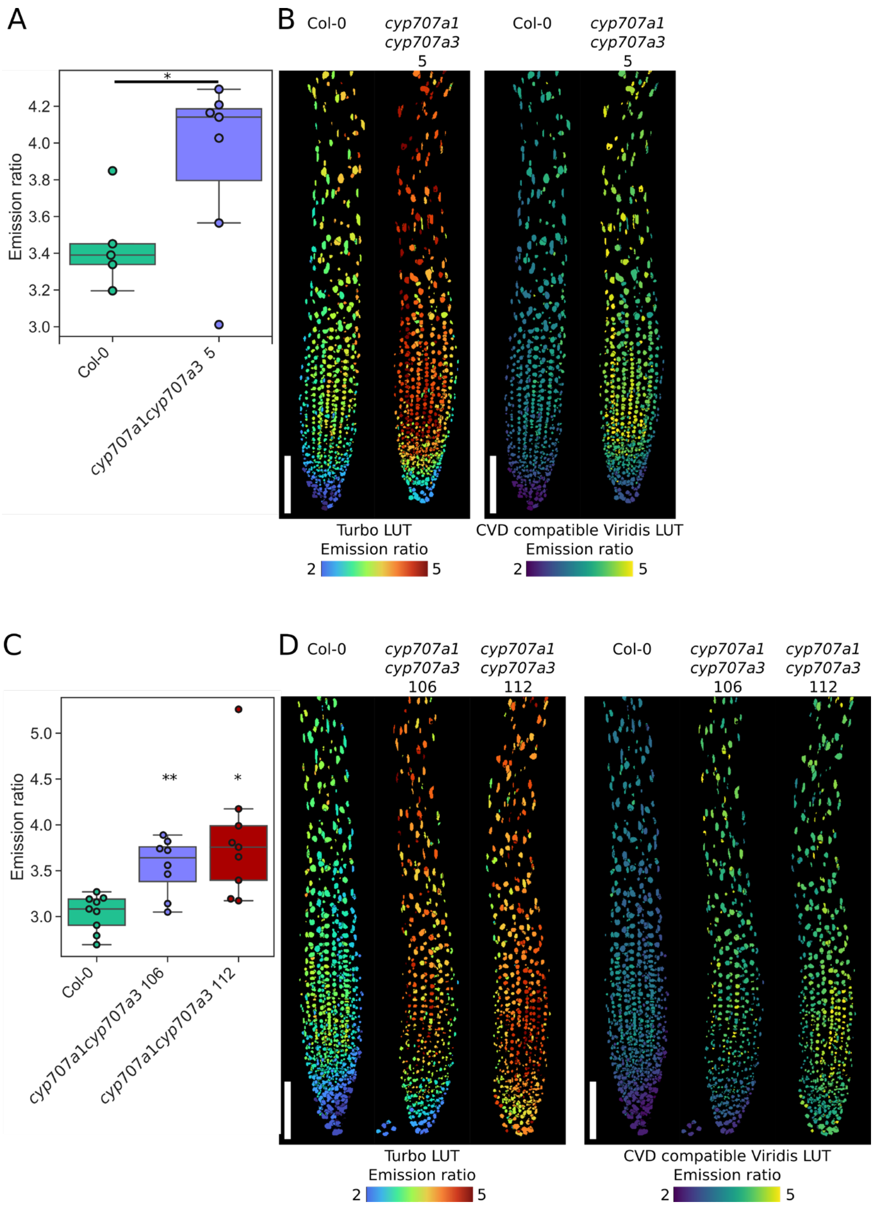

Supplementary Fig. 16. nlsABACUS2s demonstrate that *cyp707a1cyp707a3* hyperaccumulates ABA in roots.

A) and B) nlsABACUS2-100n and plants display higher emission ratios in the catabolism double mutant *cyp707a1cyp707a3* implying that these genes are essential in unstressed conditions to prevent ABA overaccumulation. An unpaired two-tailed Welsh's T test was used ($t=2.276$, $df=9.433$, $p=0.046$, $n=5,7$ biologically independent roots respectively).

C) and D) nlsABACUS2-400n and plants display higher emission ratios in the catabolism double mutant *cyp707a1cyp707a3* implying that these genes are essential in unstressed conditions to prevent ABA overaccumulation. A Brown Forsythe ANOVA was used ($p=0.0053$, $F=7.9$, $DF=11.58$) and a two-tailed Dunnett's T3 test was used to compare groups to Col-0. (line 106: $p=0.0037$, $t=3.959$, $DF=11.67$; line 112: $p=0.0108$ $t= 3.518$, $DF=9.549$; $n=9,8,9$ biologically independent roots respectively)

Scale bars indicate 100µm. For boxplots, centre line indicates median; box limits indicate upper and lower quartiles; whiskers indicate the upper/lower adjacent values. *:p<0.05, **:p<0.01, ***:p<0.001, ****:p<0.0001.

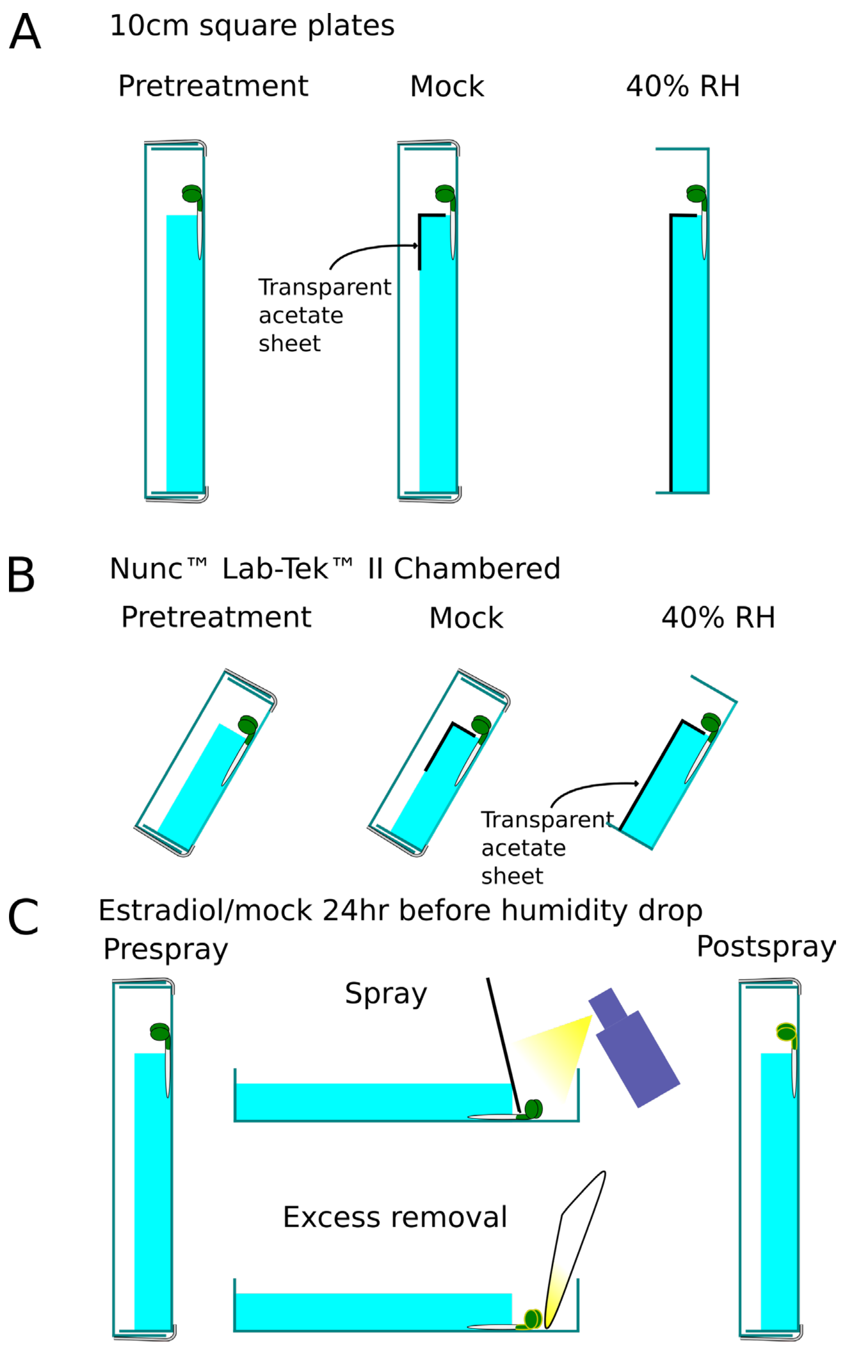

Supplementary Fig. 17. Aerial humidity treatments with hydrated roots for root growth assays and imaging assays.

A) Root growth assay where plates are opened and a piece of folded acetate sheet (thick black line) is used to cover all or part of the agar (blue). Mock plates are resealed and all plates are placed back in a growth chamber set to 40% RH.
B) Root imaging assay, where chambered coverglasses are opened and a piece of folded acetate sheet is used to cover all or part of the agar. Mock chambered coverglasses are resealed and all chambered coverglasses are placed back in a growth chamber set to 40% RH.
C) 50 µM β-estradiol treatment regime 24hr before humidity treatment for root growth assays.

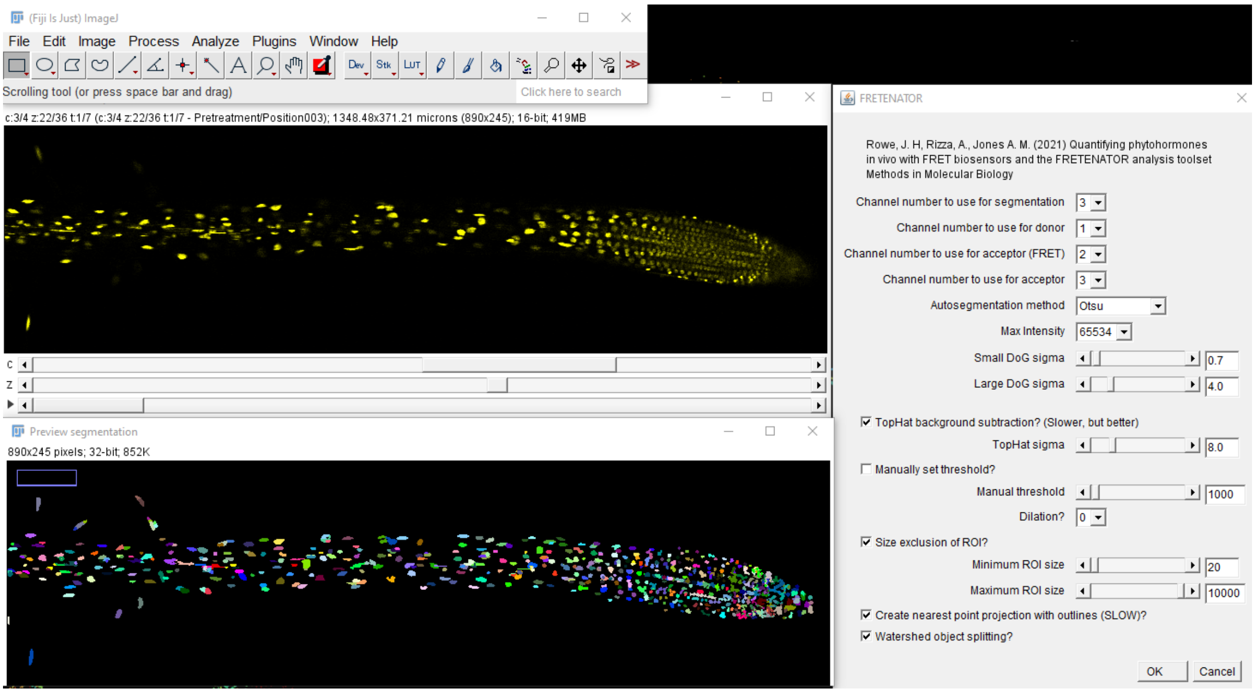

Supplementary Fig. 18. *FRETENATOR segment and ratio 1.5* user interface.

The user interface of *FRETENATOR-Segment and ratio 1.5* FIJI plugin. Various dropdowns, sliders and checkboxes on the plugin window (right) can be used to alter the segmentation settings, which will update the segmentation preview (bottom left). The Plugin window also allows channel selection for ratio calculations.

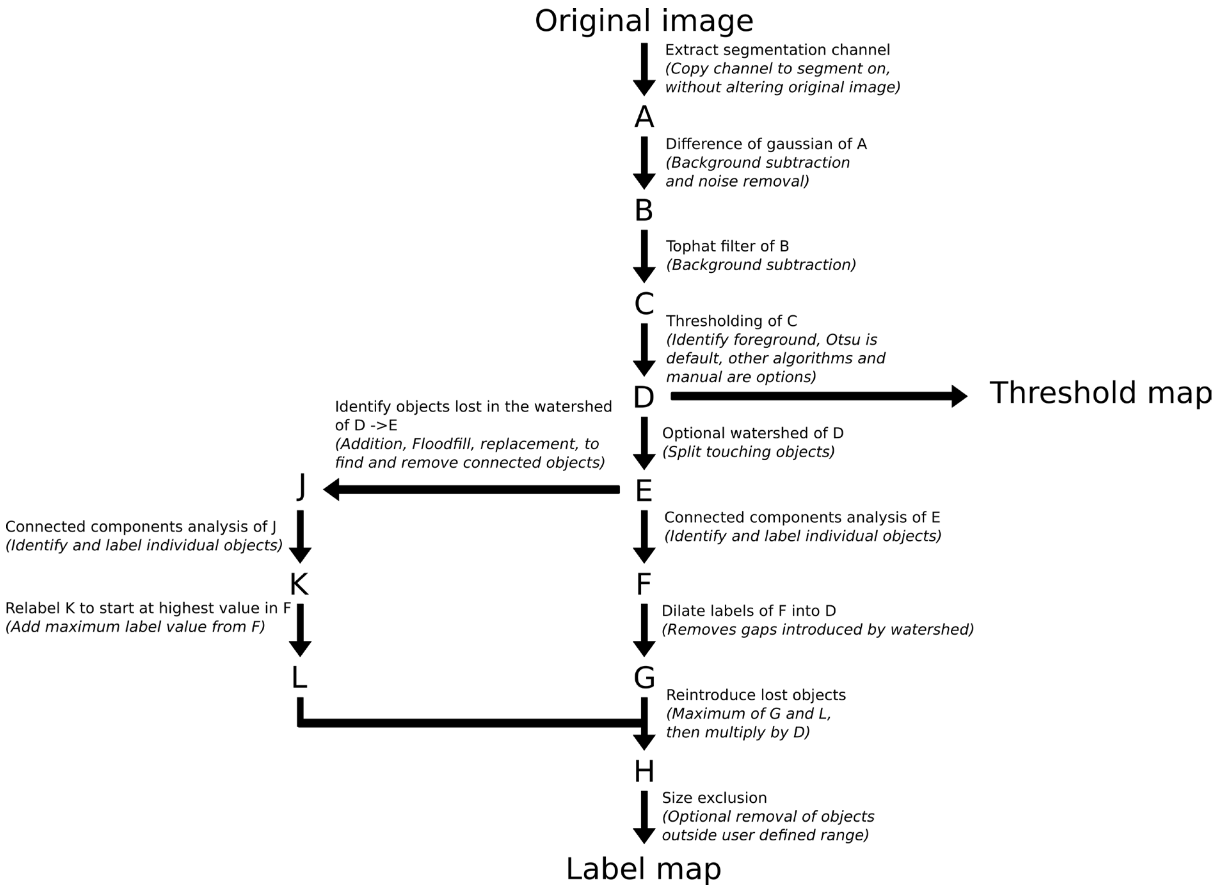

Supplementary Fig. 19. *FRETENATOR segment and ratio 1.5* segmentation pipeline.

Segmentation steps used in the *FRETENATOR-Segment and ratio 1.5* FIJI plugin. Letters indicate intermediate images used in downstream processing. Arrows indicate processing steps.

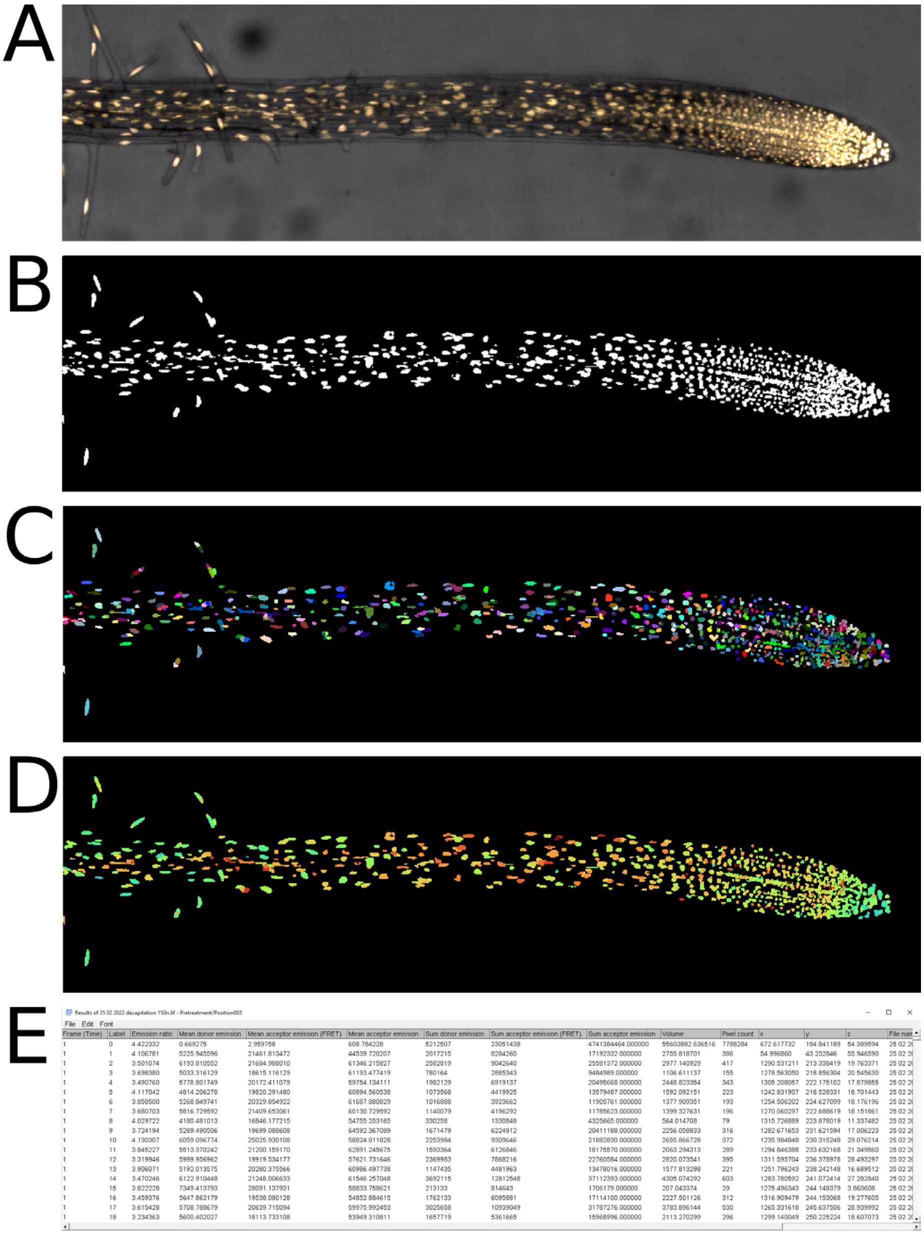

Frame (Time)	Label	Emission ratio	Mean donor emission	Mean acceptor emission (FRET)	Mean acceptor emission	Sum donor emission	Sum acceptor emission (FRET)	Sum acceptor emission	Volume	Pixel count	x	y	z	File nam
1	0	4.422332	0.669275	2.959758	608.784228	5212507	23051438	4741384464.000000	55603882.636516	7788284	672.617732	184.841189	54.389594	25 02 2
1	1	4.106781	5225.945596	21461.813472	44539.720207	2017215	8284260	17192332.000000	2755.818701	386	54.996860	43.232846	55.946590	25 02 2
1	2	3.501074	6193.810552	21684.988010	61346.215827	2582819	9042640	25581372.000000	2977.140929	417	1290.531211	213.338419	19.763371	25 02 2
1	3	3.698380	5033.316129	18615.116129	61193.477419	780164	2885343	9484989.000000	1106.611137	155	1278.563050	218.856304	20.545630	25 02 2
1	4	3.490760	5778.801749	20172.411079	59754.134111	1982129	6919137	20495668.000000	2448.823354	343	1305.208057	222.175102	17.879855	25 02 2
1	5	4.117042	4814.206278	19820.291480	60894.560538	1073568	4419925	13579487.000000	1592.092151	223	1242.831907	218.528331	18.701443	25 02 2
1	6	3.858500	5268.849741	20329.854922	61687.880829	1016888	3923662	11905761.000000	1377.909351	193	1254.506202	224.627099	18.176196	25 02 2
1	7	3.680703	5816.729592	21409.653061	60130.729592	1140079	4196292	11785623.000000	1399.327631	196	1270.060297	222.688619	18.151861	25 02 2
1	8	4.029722	4180.481013	16846.177215	54755.253165	330258	1330848	4325665.000000	564.014708	79	1315.726889	223.878019	11.337482	25 02 2
1	9	3.724194	5289.490506	19699.088608	64592.367089	1671479	6224912	20411188.000000	2256.058833	316	1282.671653	231.621594	17.006223	25 02 2
1	10	4.130307	6059.096774	25025.930108	58824.811828	2253984	9309646	21882830.000000	2655.866728	372	1235.984848	230.315248	29.076214	25 02 2
1	11	3.845227	5513.370242	21200.159170	62891.245675	1593364	6126846	18175570.000000	2063.294313	289	1294.846388	233.632168	21.349860	25 02 2
1	12	3.319946	5999.956962	19919.534177	57621.731646	2369983	7868216	22760584.000000	2820.073541	395	1311.595704	236.378978	28.493297	25 02 2
1	13	3.906071	5192.013575	20280.375566	60986.497738	1147435	4481963	13478016.000000	1577.813298	221	1251.796243	238.242148	16.689512	25 02 2
1	14	3.470246	6122.910448	21248.006633	61546.257048	3692115	12812548	37112393.000000	4305.074292	603	1283.780592	241.072414	27.282840	25 02 2
1	15	3.822228	7349.413793	28091.137931	58833.758621	213133	814643	1706179.000000	207.043374	29	1275.496343	244.148379	3.860608	25 02 2
1	16	3.459376	5647.862179	19538.080128	54852.884615	1762133	6095881	17114100.000000	2227.501126	312	1316.909479	244.153068	19.277605	25 02 2
1	17	3.615428	5708.788679	20639.715094	59975.992453	3025658	10939049	31787276.000000	3783.896144	530	1265.331618	245.637506	28.939992	25 02 2
1	18	3.234363	5600.402027	18113.733108	53949.310811	1657719	5361665	15968996.000000	2113.270299	296	1299.140049	250.225224	18.607073	25 02 2

Supplementary Fig. 20. Original image and *FRETENATOR-Segment and ratio 1.5* outputs

A) Typical import image stack, with at least two channels to calculate ratios.
B) 3D/4D stack of the thresholded image *FRETENATOR* output where foreground is assigned 1 and background is assigned 0
C) 3D/4D 'Label map' *FRETENATOR* output where each nucleus is given a unique identifier value (Displayed with the Glasbey on Dark LUT)
D) 3D/4D emission ratio map *FRETENATOR* output where each nucleus is assigned the calculated emission ratio X1000. *FRETENATOR* will also output a maximum Z-projection and outlined nearest point Z-projection of the emission ratio stack. NB: To halve the file size of exported images, emission ratio values are multiplied by 1000 in exported image files, allowing the files to be saved as 16-bit images (instead of 32-bit float images).
E) A new results table *FRETENATOR* output which can be saved as a .csv. This details the measurements of each nucleus (centroid position, size, DxDm, DxAm, AxAm, pixel count, image frame, file name, ROI identifiers).

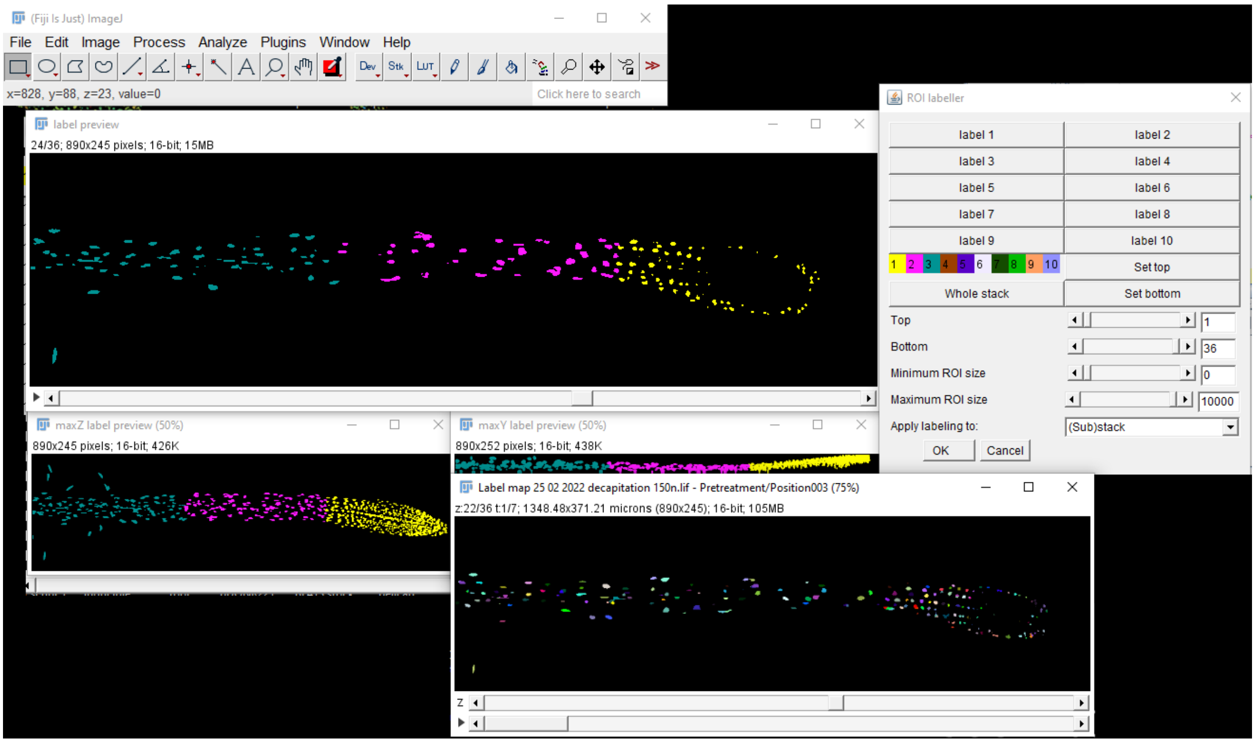

Supplementary Fig. 21. User interface of *FRETENATOR-ROI labeller.*

ImageJ selection tools (e.g. box, ellipses, line etc.) can be used to select parts of the label preview (top left) and then assign them specific labels by clicking the appropriate button on the user plugin menu panel (right). This will automatically update the preview windows.

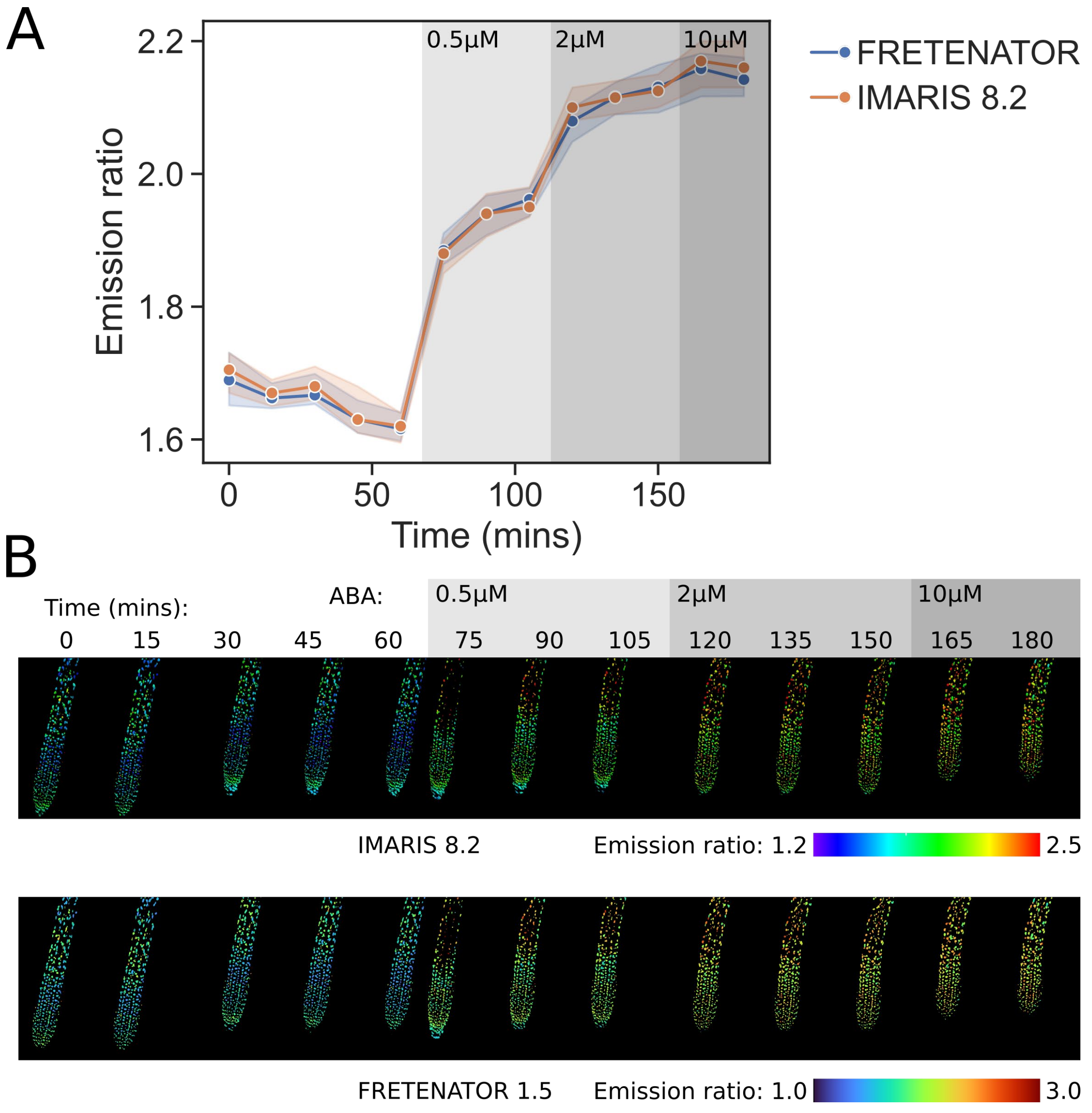

Supplementary Fig. 22. FRETENATOR 1.5 and Imaris 8.2 give similar segmentation and results

A) Quantification of emission ratios of FRETENATOR 1.5 and IMARIS 8.2 shows the same trend when quantifying an nlsABACUS2-100n ABA treatment. Line indicates median, shaded area indicates 95% confidence interval.

B) FRETENATOR 1.5 and IMARIS 8.2 show similar segmentation and emission ratio patterns when quantifying an nlsABACUS2-100n ABA treatment.

Note this experiment is a software validation, and so uses the same dataset as Supplementary Fig. 6.

Tables

Supplemental Table 1. Emission ratios of selected purified ABACUS variants *in vitro*.

Sensor	AVERAGE K_D (µM)	SD K_D (µM)	AVERAGE RC (%)	SD RC	n
**ABACUS1-2µ PYL1 H87P<br>Linkers: attB1-L52-attB2<br>FP: edCit, edCer**	**1.143**	**0.499**	**43.0**	**5.0**	**6**
**ABACUS1-2µ-i<br>PYL1 H87P, A190V<br>Linkers: attB1-L52-attB2<br>FP: edCit, edCer**	**0.911**	**0.047**	**30.3**	**4.7**	**3**
**ABACUS1-2µ-ii<br>PYL1 H87P, A190V<br>Linkers: PPP-L52-P<br>FP: edCit, edCer**	**0.948**	**0.460**	**63.3**	**14.7**	**12**
**ABACUS1-2µ -iii<br>PYL1 H87P, A190V, S112A<br>Linkers: PPP-L52-P<br>FP: edCit, edCer**	**0.578**	**0.300**	**68.9**	**15.5**	**5**
**ABACUS1-2µ-iv<br>PYL1 H87P, A190V, S112A<br>Linkers: PPP-L52-P;<br>FP: edCitT9, T7edCer**	**0.472**	**0.231**	**52.3**	**10.4**	**15**
**ABACUS2-400n<br>PYL1 H87P, A190V, S112A, R143S<br>Linkers: PPP-L52-P<br>FP: edCitT9, T7edCer**	**0.445**	**0.222**	**71.0**	**19.2**	**16**
**ABACUS2-100n<br>PYL1 H87P, A190V, S112A, E141D<br>Linkers: PPP-L52-P<br>FP: edCitT9, T7edCer**	**0.098**	**0.037**	**67.6**	**25.8**	**13**

Supplemental Table 2. Additional ABACUS variant screening

(Attached as an Excel file)

Supplemental Table 3. *Arabidopsis* germplasm used in this study

Line	Background	Creation method	Origin	Selection	Approx % homozygous seed batches that are fluorescent *	NASC ID
Col-0			NASC			
UBQ10pro::nlsABACUS2-400n line 1	Col-0	Floral dip	This study	FAST-RED	ND	TBD
UBQ10pro::nlsABACUS2-400n line 7	Col-0	Floral dip	This study	FAST-RED	~38	TBD
UBQ10pro::nlsABACUS2-100n line 7	Col-0	Floral dip	This study	FAST-RED	~17	TBD
UBQ10pro::nlsABACUS2-100n line 18	Col-0	Floral dip	This study	FAST-RED	ND	TBD
P16pro::nlsABACUS1-2µ	Col-0	Floral dip	This study		~70	TBD
UBQ10pro:XVE>>NCED3 line 33	Col-0	Floral dip	This study	Hygromycin	N/A	TBD
UBQ10pro:XVE>>CYP707A3 line 99	Col-0	Floral dip	This study	Hygromycin	N/A	TBD
SUC2pro:XVE>> CYP707A3 line 194	Col-0	Floral dip	This study	Hygromycin	N/A	TBD
UBQ10pro:XVE>>NCED3 UBQ10pro::nlsABACUS2-100n line 1	UBQ10pro:XVE>>NCED3 line 33	Floral dip	This study	Hygromycin, FAST-RED	Not homozygous for ABACUS	TBD
UBQ10pro:XVE>>NCED3 UBQ10pro::nlsABACUS2-400n line 6	UBQ10pro:XVE>>NCED3 line 33	Floral dip	This study	Hygromycin, FAST-RED	Not homozygous for ABACUS	TBD
UBQ10pro:XVE>>CYP707A3 UBQ10pro::nlsABACUS2-100n line 1	UBQ10pro:XVE>>CYP707A3 line 99	Floral dip	This study	Hygromycin, FAST-RED	Not homozygous for ABACUS	TBD
UBQ10pro:XVE>> CYP707A3 UBQ10pro::nlsABACUS2-400n line 2	UBQ10pro:XVE>>CYP707A3 line 99	Floral dip	This study	Hygromycin, FAST-RED	Not homozygous for ABACUS	TBD
snrk2.2snrk2.3	Col-0	-	[50]	-	N/A	
snrk2.2snrk2.3 RCH1pro::SnRK2.2	*snrk2.2snrk2.3*	-	[50]	-	N/A	
snrk2.2snrk2.3 SnRK2.2pro::SnRK2.2	*snrk2.2snrk2.3*	-	[50]	-	N/A	
aba2-1	Col-0	-	[73]	-	N/A	N156
cyp707a1cyp707a3	Col-0	-	[39]	-	N/A	
cyp707a1cyp707a3 UBQ10pro::nlsABACUS2-400n – line 106	*cyp707a1cyp707a3*	Floral dip	This study	FAST-RED	Not homozygous for ABACUS	TBD
cyp707a1cyp707a3 UBQ10pro::nlsABACUS2-400n - line 112	*cyp707a1cyp707a3*	Floral dip	This study	FAST-RED	Not homozygous for ABACUS	TBD
cyp707a1cyp707a3 UBQ10pro::nlsABACUS2-100n line 5	*cyp707a1cyp707a3*	Floral dip	This study	FAST-RED	Not homozygous for ABACUS	TBD

*Note on silencing: Newly germinated plants from an unsilenced parent express the sensor strongly in most seedlings. However, silencing often occurs after seedling establishment and we encourage growers to bulk plants individually, expecting 10-40% of

seed batches to have fluorescence. For genetic crosses, screening flowering ABACUS2 plants for fluorescence at the point of performing the cross consistently leads to fluorescent F1 seedlings.

Supplemental Table 4. Primers used in this study

SDM primers for ABACUS variants:

PYL1 QuikChange Primer	Sequence
A190V Fw	gattcaatctaataaccgtatcaacaaaacaatctcgtatcttcctcc
A190V Rv	ggaggaagatacgagttgttttgttgatacggttatagattgaatc
S112A Fw	ccggtaatccagctatcacgttcacgtcgcgcgt
S112A Rv	acgcgcgacgtgaacgtgatagctggattaccgg
E141D Fw	gtttagtataaccggtggtgatcataggctgaggaattataaa
E141D Rv	tttataattcctcagcctatgatcaccaccggttatactaaac
R143S Fw	accggtggtgaacatagcctgaggaattataaatcg
R143S Rv	cgatttataattcctcaggctatgttcaccaccggt
K90A Fw	cacagatttacaaacccttcatcgcaagctgtaacgtgagtgaagatt
K90A Rv	aatcttcactcacgttacagcttgcgatgaagggtttgtaaatctgtg
T138I Fw	gagagtgactgggtttagtataatcggtggtgaaca
T138I Rv	tgttcaccaccgattatactaaacccagtcactctc
F189L Fw	ggaggaagatacgagattgttagttgatacggttattagattg
F189L Rv	caatctaataaccgtatcaactaacaatctcgtatcttcctcc
I89S Fw	acagatttacaaacccttcagcaaaagctgtaacgtgagtg
I89S Rv	cactcacgttacagcttttgctgaagggtttgtaaatctgt
S182A Fw	tgttgttgatgtaccggaaggtaatgcggaggaagatac
S182A Rv	gtatcttcctccgcattaccttccggtacatcaacaaca
S136A Fw	cggagagtgactgggtttgctataaccggtggtgaaca
S136A Rv	tgttcaccaccggttatagcaaacccagtcactctccg
I194V Fw	cgagattgtttgttgatacggttgttagattgaatcttcagaaactt
I194V Rv	aagtttctgaagattcaatctaacaaccgtatcaacaaacaatctcg
I111K Fw	acgcgcgacgtgaacgtgaaagccggatta
I111K Rv	taatccggctttcacgttcacgtcgcgcgt
F135I Fw	tcggagagtgactgggattagtataaccggtgg
F135I Rv	ccaccggttatactaatcccagtcactctccga
F189I Fw	cggaggaagatacgagattgattgttgatacggttattaga
F189I Rv	tctaataaccgtatcaacaatcaatctcgtatcttcctccg
K90R Fw	gatttacaaacccttcatcagaagctgtaacgtgagtgaag
K90R Rv	cttcactcacgttacagcttctgatgaagggtttgtaaatc
K90S Fw	ggccacagatttacaaacccttcatcagcagctgtaacgtgag
K90S Rv	ctcacgttacagctgctgatgaagggtttgtaaatctgtggcc
I111G Fw	cgcgacgtgaacgtgggagctggattaccggc
I111G Rv	gccggtaatccagctcccacgttcacgtcgcg
V193L Fw	gatacgagattgtttgttgatacgcttattagattgaatcttcagaaac
V193L Rv	gtttctgaagattcaatctaataagcgtatcaacaaacaatctcgtatc
F189L Fw	ggaggaagatacgagattgttagttgatacggttattagattg
F189L Rv	caatctaataaccgtatcaactaacaatctcgtatcttcctcc
E171Q Fw	ggatctggaccgttgttttgcagtcttatgttgttgatgtacc
E171Q Rv	ggtacatcaacaacataagactgcaaaacaacggtccagatcc
G113P Fw	gacgtgaacgtgatagctccattaccggcgaatacgtc
G113P Rv	gacgtattcgccggtaatggagctatcacgttcacgtc

T138I Fw	gagagtgactgggtttagtataatcggtggtgaaca
T138I Rv	tgttcaccaccgattatactaaacccagtcactctc

Linker swap, fluorescent protein truncation and Citrine codon diversification primers:

In-Fusion Primer	Sequence
Cit 3' ABI1aid 5' Fwd	ggacgagctgtacaagcataaaccggatagagaagatga
edCit 3' plus 1 proline ABI1aid 5' Fwd	ggacgagctgtacaagccacataaaccggatagagaagatga
edCit 3' plus 2 prolines ABI1aid 5' Fwd	ggacgagctgtacaagccacctcataaaccggatagagaagatga
edCit 3' plus 3 prolines ABI1aid 5' Fwd	ggacgagctgtacaagccacctccacataaaccggatagagaagatga
edCer 5' Rev PYL1 3' Rev	cctcgcccttgctcaccctaacctgagaagagttgttgt
edCer 5' Rev plus 1 proline PYL1 3' Rev	cctcgcccttgctcacaggcctaacctgagaagagttgttgt
edCer 5' Rev plus 2 prolines PYL1 3' Rev	cctcgcccttgctcacaggtggcctaacctgagaagagttgttgt
edCer 5' Rev plus 3 prolines PYL1 3' Rev	cctcgcccttgctcacaggtggaggcctaacctgagaagagttgttgt
edCit Codon Diversified-T9 fw	ccacctccacataaaccgga
edCit Codon Diversified-T9 rv	tttatgtggaggtggtataccagcagcagt
T7-edCer fw	tctcaggttaggcctttcaccggggtggtg
T7-edCer rv	aggcctaacctgagaagagt

Primers to generate gateway constructs and replace codon diversified Citrine:

	Name	Sequence	Amplified with	Description
1	NCED3 CDS F1 attB1	GGGG ACA AGT TTG TAC AAA AAA GCA GGC TTT atg gct tct ttc acg gca ac	2	Multisite gateway
2	NCED3 CDS R1 attB2	GGGG AC CAC TTT GTA CAA GAA AGC TGG GT tca cac gac ctg ctt cg	1	Multisite gateway
3	pDONR backbone F	GATTTATAATAGACCCAGCTTTCTTG TACAAAG	4	infusion cloning of ABACUS2 non codon diversified edCitrine
4	pDONR backbone NLS R	ACCTCGAGCCCTCCAACCTTTCTCTTC TTC	3	infusion cloning of ABACUS2 non codon diversified edCitrine
5	Start NLSedCit F	TTGGAGGGCTCGAGGTGAGCAAGGG	6	infusion cloning of ABACUS2 non codon diversified edCitrine
6	edCIT R	GGAGGTGGCTTGTACAGCTCGTCCAT GC	5	infusion cloning of ABACUS2 non codon diversified edCitrine
7	ABI1aid F	TGTACAAGCCACCTCCACATAAACCG GATA	8	infusion cloning of ABACUS2 non codon diversified edCitrine
8	myc STOP R	CTGGGTCTATTATAAATCTTCCTCACT TATC	7	infusion cloning of ABACUS2 non codon diversified edCitrine
