# Supplementary material for: Next-generation ABACUS biosensors reveal cellular ABA dynamics driving root growth at low aerial humidity
Source: Nat Plants. 2023 Jun 26;9(7):1103–15. doi: 10.1038/s41477-023-01447-4 (PMC10356609; doi:10.1038/s41477-023-01447-4)
Supplement: Supplementary file 2 — Reporting Summary [file 41477_2023_1447_MOESM2_ESM.pdf]

nature portfolio

Corresponding author(s): Alexander M Jones

Last updated by author(s): May 12, 2023

Reporting Summary

Nature Portfolio wishes to improve the reproducibility of the work that we publish. This form provides structure for consistency and transparency in reporting. For further information on Nature Portfolio policies, see our Editorial Policies and the Editorial Policy Checklist.

Statistics

For all statistical analyses, confirm that the following items are present in the figure legend, table legend, main text, or Methods section.

n/a	Confirmed	
☐	☒	The exact sample size (*n*) for each experimental group/condition, given as a discrete number and unit of measurement
☐	☒	A statement on whether measurements were taken from distinct samples or whether the same sample was measured repeatedly
☐	☒	The statistical test(s) used AND whether they are one- or two-sided<br>*Only common tests should be described solely by name; describe more complex techniques in the Methods section.*
☐	☒	A description of all covariates tested
☐	☒	A description of any assumptions or corrections, such as tests of normality and adjustment for multiple comparisons
☐	☒	A full description of the statistical parameters including central tendency (e.g. means) or other basic estimates (e.g. regression coefficient) AND variation (e.g. standard deviation) or associated estimates of uncertainty (e.g. confidence intervals)
☐	☒	For null hypothesis testing, the test statistic (e.g. *F*, *t*, *r*) with confidence intervals, effect sizes, degrees of freedom and *P* value noted<br>*Give P values as exact values whenever suitable.*
☒	☐	For Bayesian analysis, information on the choice of priors and Markov chain Monte Carlo settings
☒	☐	For hierarchical and complex designs, identification of the appropriate level for tests and full reporting of outcomes
☒	☐	Estimates of effect sizes (e.g. Cohen's *d*, Pearson's *r*), indicating how they were calculated

Our web collection on statistics for biologists contains articles on many of the points above.

Software and code

Policy information about availability of computer code

Data collection	Leica LAS X was used to perform confocal microscopy. Spectramax softmax pro was used for biosensor fluorescence spectroscopy. EPSON Perfection V700 Photo scanner was used for root phenotype scanning.
Data analysis	Most data analysis was performed using well documented, publicly available code libraries, such as Python, Matplotlib and Seaborn. MS Excel and Graphpad Prism were used for other some analysis as detailed in the text. The custom analysis pipelines (FRETENATOR1.5) are open source and detailed on the github detailed in the text. IMARIS 8.2 was used as a comparator to validate FRETENATOR. Fiji 1.53 was used for other image analysis. LabVIEW was used as the SPIM software.

For manuscripts utilizing custom algorithms or software that are central to the research but not yet described in published literature, software must be made available to editors and reviewers. We strongly encourage code deposition in a community repository (e.g. GitHub). See the Nature Portfolio guidelines for submitting code & software for further information.

Data

Policy information about availability of data

All manuscripts must include a data availability statement. This statement should provide the following information, where applicable:

- Accession codes, unique identifiers, or web links for publicly available datasets
- A description of any restrictions on data availability
- For clinical datasets or third party data, please ensure that the statement adheres to our policy

New plant lines will be deposited at the Nottingham Arabidopsis Stock Centre. Binary vectors for ABACUS2 transformation as plant ABACUS2 constructs in pENTR221-f1 will be deposited at Addgene. All data will be placed in the Cambridge data repository https://doi.org/10.17863/CAM.96615. The FRETENATOR image analysis toolset, as well as installation and usage instructions are available at https://github.com/JimageJ/ImageJ-Tools.

Human research participants

Policy information about studies involving human research participants and Sex and Gender in Research.

Reporting on sex and gender	*Use the terms sex (biological attribute) and gender (shaped by social and cultural circumstances) carefully in order to avoid confusing both terms. Indicate if findings apply to only one sex or gender; describe whether sex and gender were considered in study design whether sex and/or gender was determined based on self-reporting or assigned and methods used. Provide in the source data disaggregated sex and gender data where this information has been collected, and consent has been obtained for sharing of individual-level data; provide overall numbers in this Reporting Summary. Please state if this information has not been collected. Report sex- and gender-based analyses where performed, justify reasons for lack of sex- and gender-based analysis.*
Population characteristics	*Describe the covariate-relevant population characteristics of the human research participants (e.g. age, genotypic information, past and current diagnosis and treatment categories). If you filled out the behavioural & social sciences study design questions and have nothing to add here, write "See above."*
Recruitment	*Describe how participants were recruited. Outline any potential self-selection bias or other biases that may be present and how these are likely to impact results.*
Ethics oversight	*Identify the organization(s) that approved the study protocol.*

Note that full information on the approval of the study protocol must also be provided in the manuscript.

Field-specific reporting

Please select the one below that is the best fit for your research. If you are not sure, read the appropriate sections before making your selection.

☒ Life sciences ☐ Behavioural & social sciences ☐ Ecological, evolutionary & environmental sciences

For a reference copy of the document with all sections, see nature.com/documents/nr-reporting-summary-flat.pdf

Life sciences study design

All studies must disclose on these points even when the disclosure is negative.

Sample size	Sample size was determined empirically, based on typical sample sizes for similar work.
Data exclusions	During imaging, some samples were excluded due to handling mistakes or technical errors (e.g. crushed plant samples when mounting). For root length assays, arrested roots and plants that germinated late were excluded before analysis. No data were excluded after analysis.
Replication	All experiments were repeated at least twice with the similar results.
Randomization	Although samples weren't randomised as such, plates/sample growth chambers were alternated in the growth chambers, to minimise location dependent effects. During treatments, sample types were typically staggered and alternated (e.g. imaging 2-3 mock samples followed by 2-3 treatment samples then 2-3 mock samples etc) to minimise any time dependent effects.
Blinding	Blinding was not performed as image analysis was mostly automated, and so would have no effect on the results.

Reporting for specific materials, systems and methods

We require information from authors about some types of materials, experimental systems and methods used in many studies. Here, indicate whether each material, system or method listed is relevant to your study. If you are not sure if a list item applies to your research, read the appropriate section before selecting a response.

Materials & experimental systems

n/a	Involved in the study
☒	☐ Antibodies
☒	☐ Eukaryotic cell lines
☒	☐ Palaeontology and archaeology
☒	☐ Animals and other organisms
☒	☐ Clinical data
☒	☐ Dual use research of concern

Methods

n/a	Involved in the study
☒	☐ ChIP-seq
☒	☐ Flow cytometry
☒	☐ MRI-based neuroimaging
